# Towards the total synthesis of chondrochloren A: synthesis of the (*Z*)-enamide fragment

**DOI:** 10.3762/bjoc.16.64

**Published:** 2020-04-14

**Authors:** Jan Geldsetzer, Markus Kalesse

**Affiliations:** 1 Helmholtz Centre of Infection Research (HZI), Inhoffenstr. 7, 38124 Braunschweig, Germany; 2 Leibniz Universität Hannover, Institute of Organic Chemistry, Schneiderberg 1B, 30167 Hannover, Germany,; 3 Centre for Biomolecular Drug Research (BWMZ), Schneiderberg 38, 30167 Hannover, Germany

**Keywords:** cross coupling, myxobacteria, natural product, ribolactone, Z-enamide

## Abstract

The stereoselective synthesis of the (*Z*)-enamide fragment of chondrochloren (**1**) is described. A Buchwald-type coupling between amide **3** and (*Z*)-bromide **4** was used to generate the required fragment. The employed amide **3** comprising three chiral centers was obtained through a seven-step sequence starting from ᴅ-ribonic acid-1,4-lactone. The (*Z*)-vinyl bromide **4** is accessible in four steps from 4-hydroxybenzaldehyde. The pivotal cross coupling between both fragments was achieved after extensive experimentation with copper(I) iodide, K_2_CO_3_ and *N*,*N*′-dimethylethane-1,2-diamine.

## Introduction

In the course of our program to provide synthetic access to biologically active natural products we targeted complex polyketides and depsipetides [1–10]. One particular group of compounds of particular focus in our research activities are natural products with enamide moieties [11]. Among these, chondrochloren having a (*Z*)-enamide moiety features a rare structural motif. The myxobacterial metabolite chondrochloren A (**1**) was isolated from *Chondromyces crocatus* (Cmc5) by the groups of Höfle and Reichenbach in 2003 [12]. This PKS/NRPS-derived natural product shows only weak antibiotic effects in agar diffusion tests against *Micrococcus luteus*, S*chizosaccharomyces pombe*, *Bacillus subtilis* and *Staphylococcus aureus* [12]. The relative and absolute stereochemistry of the molecule was elucidated by a combination of NMR, UV and IR spectroscopy and molecular dynamics calculations (MD, MM2) [12]. However, its (*Z*)-enamide motif and the polyoxygenated middle segment are synthetically challenging.

## Results and Discussion

### Synthesis of amide **3**

**Figure 1:**
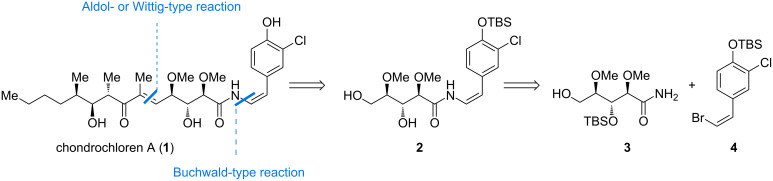
Retrosynthetic analysis of chondrochlorene A (**1**).

Here we report our investigations on the construction of the segments **3** and **4** as well as our results on the cross coupling between both fragments. Our retrosynthetic approach (Figure 1) divides chondrochloren A (**1**) into three fragments of similar complexity: amide **2**, (*Z*)-bromide **4** and amide **3**. For the coupling of amide **3** and (*Z*)-bromide **4** we decided to use a (*Z*)-selective Buchwald-type reaction encouraged by the previous works of the Buchwald group on producing (*E*)-enamide coupling products [13–15].

**Scheme 1:**
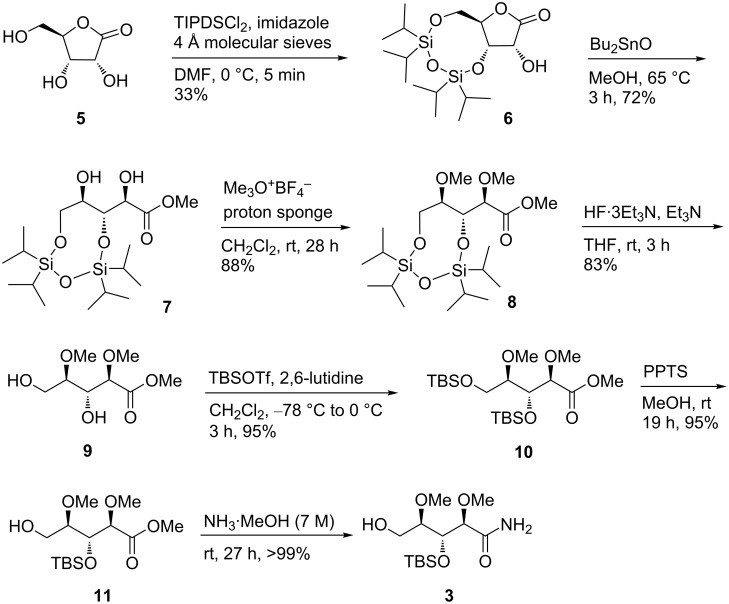
Synthesis of amide **3** [16–20]. TIPDSCl_2_ = 1,3-dichloro-1,1,3,3-tetraisopropyldisiloxane, TBSOTf = *tert*-butyldimethylsilyl trifluoromethanesulfonate, proton sponge = 1,8-bis(*N*,*N*-dimethylamino)naphthalene.

The synthesis of the amide **3** started with TIPDS-protection of commercially available ᴅ-ribonic acid-1,4-lactone (**5**) (Scheme 1). A subsequent transesterification under mild conditions with Bu_2_SnO provided dihydroxy ester **7** in 72% yield. The 1,3-diol in **7** was methylated with an excess of the Meerwein reagent and TIPDS-removal afforded ester **9** in good yields. A double TBS-protection and liberation of the primary alcohol provided alcohol **11** in an excellent yield which was subjected to aminolysis to provide amide **3** in seven steps and an overall yield of 16% [16–20].

### Synthesis of (*Z*)-bromide **4**

**Scheme 2:**
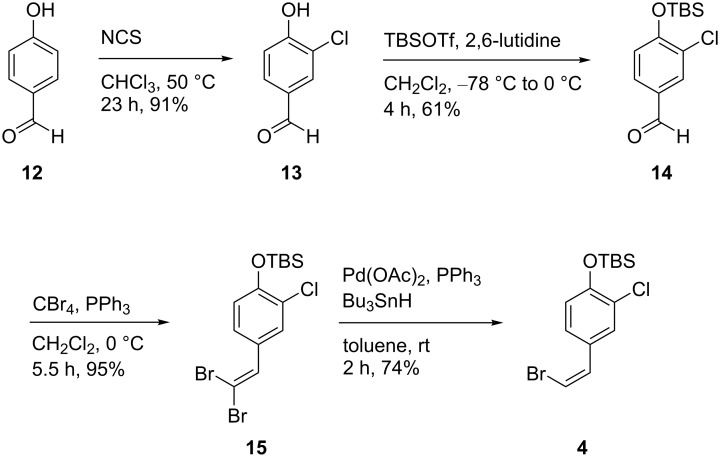
Synthesis of (*Z*)-bromide **4** using a palladium-catalyzed, stereoselective dehalogenation [21–24]. TBSOTf = *tert*-butyldimethylsilyl trifluoromethanesulfonate, NCS = *N*-chlorosuccinimide, 2,6-lutidine = 2,6-dimethylpyridine.

For the synthesis of (*Z*)-bromide **4** we chose a palladium-catalyzed, stereoselective dehalogenation as the key step (Scheme 2). Therefore, 4-hydroxybenzaldehyde (**12**) was chlorinated and phenol **13** was protected as TBS ether to afford aldehyde **14** which was then converted into dibromoolefine **15** in good yields using the Corey–Fuchs protocol. Uenishi et al. [21] published an effective way of defunctionalizing dihalogenated double bonds into the corresponding (*Z*)-monohalogenated derivatives using palladium(II) acetate, triphenylphosphine and tributyltin hydride. Following this procedure, we were able to obtain (*Z*)-bromide **4** in four steps and an overall yield of 39% [21–24].

### Cross coupling of fragments **3** and **4**

**Scheme 3:**
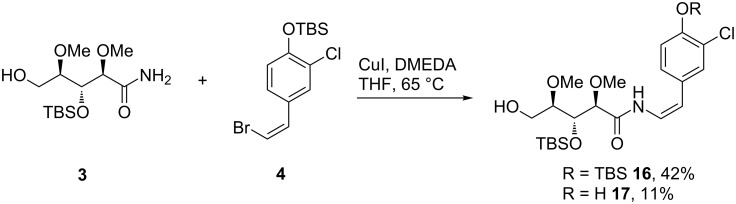
Cross coupling of amide **3** and (*Z*)-bromide **4** (see Table 1 for conditions).

The formation of the (*Z*)-enamide should occur in a copper-catalyzed Buchwald-type reaction (Scheme 3). Based on a previous work of Buchwald and his group [13], we decided to use copper(I) iodide and *N*,*N′*-dimethylethylenediamine (DMEDA) as the catalytic system in THF, which was reported to be the solvent of choice in this type of coupling reaction. Potassium carbonate was chosen due to the sensitivity of the amide **3** towards harsh basic conditions. With these conditions we were able to couple (*Z*)-bromide **4** with amide **3** selectively to yield (*Z*)-enamide **16**. The obtained double bond geometry was confirmed by the indicative NMR coupling constants of 9.6 Hz. Moreover, we observed a concentration dependent formation of the undesired desilylated (*Z*)-enamide **17** (Table 1). The best results were achieved using a 65 mM solution of the amide **3**. Using dry potassium carbonate, purified copper(I) iodide provided the best results for the cross-coupling reaction.

**Table 1:** Concentration studies of amide **3** and (*Z*)-bromide **4** in the Buchwald-type reaction.^a^

entry	*c* (amide **3**, mM)	**16**	**17**	**3**

1	20	33	–	31
2	65	42	11	–
3	136	3	11	2
4	140	17	27	–

^a^Conditions: amide **3** (1.0 equiv), (*Z*)-bromide **4** (1.0 equiv), DMEDA (0.60 equiv), CuI (0.30 equiv), THF, 65 °C. DMEDA = *N*,*N*′-dimethylethylenediamine.

## Conclusion

In summary, we established a (*Z*)-selective Buchwald-type coupling reaction as a key step in the synthesis of an advanced fragment of chondrochloren A (**1**). The required amide **3** can be synthesized in seven steps with a 16% overall yield [16–20], whereas the (*Z*)-bromide **4** can be generated in a four-step sequence with a 39% overall yield, including a palladium-catalyzed, stereoselective dehalogenation [21–25].

## Supporting Information

File 1 Experimental procedures and spectral data of the synthesized compounds.
