# Supplementary material for: Towards the total synthesis of chondrochloren A: synthesis of the (Z)-enamide fragment
Source: Beilstein J Org Chem. 2020 Apr 14;16:670–3. doi: 10.3762/bjoc.16.64 (PMC7176928; doi:10.3762/bjoc.16.64)
Supplement: File 1 — Experimental procedures and spectral data of the synthesized compounds. [file Beilstein_J_Org_Chem-16-670-s001.pdf]

BEILSTEIN JOURNAL OF ORGANIC CHEMISTRY

Supporting Information

for

Towards the total synthesis of chondrochloren A: synthesis of the (*Z*)-enamide fragment

Jan Geldsetzer and Markus Kalesse

Beilstein J. Org. Chem. **2020,** *16,* 670–673. doi:10.3762/bjoc.16.64

Experimental procedures and spectral data of the synthesized compounds

License and Terms: This is a supporting information file under the terms of the Creative Commons Attribution License (http://creativecommons.org/licenses/by/4.0). Please note that the reuse, redistribution and reproduction in particular requires that the authors and source are credited.
The license is subject to the *Beilstein Journal of Organic Chemistry* terms and conditions: (https://www.beilstein-journals.org/bjoc)

General information:

All reactions were conducted in dried flasks under argon and stirring, unless it was definitively not necessary. All solvents were used as received from Acros Organics and Sigma-Aldrich. THF was used after being distilled over Na/benzophenone under argon. Triethylamine and dichloromethane were used after distillation over CaH_2 under N_2 atmosphere. Reagents that are commercially available were used as received. Temperatures refer to oil bath temperatures. All reactions were carried out with a magnetic stirrer. After drying over either $MgSO_4$ or Na_2SO_4, the suspension was filtered before removing the solvent in vacuo.

Reactions were monitored by TLC. The TLC plates were used from Macherey-Nagel Alugram Xtra SIL G/UV_{254}, aluminum plates, silica 60. The visualization occurred by UV fluorescence (254 nm) or by staining with $KMnO_4$ (30% in H_2O). Flash column chromatography was performed with Macherey-Nagel brand silica gel (60 M, 0.04–0.063 mm mesh). The eluent is given in volume ratios (*v*/*v*).

NMR spectra were recorded on a Bruker Ultrashield 400 MHz Avance-I at room temperature. Chemical shifts δ are given in ppm relative to residual protonated solvent peaks ($CHCl_3$: δ_H = 7.26 ppm, δ_C = 77.2 ppm; DMSO: δ_H = 2.50 ppm; δ_C = 39.5 ppm; CH_3OH: δ_H = 3.35 ppm; δ_C = 49.3 ppm). The following descriptions are used for ^1H spectra: s (singlet), bs (broad singlet), d (doublet), t (triplet), q (quartett), m (multiplet) or combinations of these acronyms.

Electrospray mass spectra were recorded using either a Waters QTOF-Premier (Waters Aquity Ultra Performance, ESI) or a LCT Premier (Waters). The ionization modes, the calculated and found mass are given.

Optical rotations were obtained using a Perkin Elmer 341 polarimeter and following standard conditions: wavelength 589.3 nm (sodium D-line), cell length 1 dm, solvent and sample concentration g/100 mL are given in the individual experiment.

Synthesis of TIPDS-protected lactone 6:

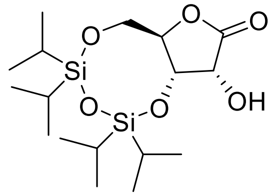

$C_{17}H_{34}O_6Si_2$
390.62 g/mol
6

(γ)-Ribonolacton (**5**, 1.17 g, 7.90 mmol, 1.00 equiv), imidazole (2.61 g, 38.3 mmol, 4.85 equiv) and molecular sieves (4 Å, 1.00 g) were dispersed in dry DMF (80.0 mL). The suspension was cooled to 0 °C. 1,3-Dichloro-1,1,3,3-tetraisopropyldisiloxane (3.0 mL, 2.90 g, 9.13 mmol, 1.15 equiv) was added dropwise and the reaction mixture was stirred for further 5 min at 0 °C. After the addition of an aqueous NH_4Cl solution (50 mL) and warming to room temperature, the layers were separated. The aqueous layer was extracted with EtOAc (3 × 100 mL). The combined organic layer was washed with water (5 × 150 mL), brine (150 mL), dried over $MgSO_4$ and concentrated in vacuo. The crude product was purified by flash column chromatography (PE/EtOAc 9:1) delivering the TIPDSi-protected lactone **6** (1.01 g, 2.59 mmol, 33%) as colorless oil.

R*f* **(**PE:EtOAc = 2:1) = 0.88;

$[\alpha]_D^{20}$ = +17.7 (*c* = 1.0, $CHCl_3$);

S. Hildbrand, A. Blaser, S. P. Parel, C. J. Leumann, *J. Am. Chem. Soc.* **1997**, *119*, 5499-5511:

$[\alpha]_D^{20}$ = +28.9, *c* = 0.5, $CHCl_3$;

^{1}H NMR (400 MHz, $CDCl_3$): δ [ppm] = 4.49 (dd, *J* = 6.9, 5.8 Hz, 1 H), 4.43 (ddd, *J* = 6.9, 5.8, 3.5 Hz, 1 H), 4.23 (d, *J* = 5.8 Hz, 1 H), 4.13 (dd, *J* = 12.6, 3.5 Hz, 1 H), 3.99 (dd, *J* = 12.6, 5.8 Hz, 1 H), 3.16 (bs, 1 H), 1.09–1.03 (m, 24 H), 0.97–0.81 (m, 4 H);

^{13}C NMR (100 MHz, $CDCl_3$): δ [ppm] = 172.2, 82.2, 69.9, 68.8, 61.6, 17.5, 17.4, 17.3, 17.3, 17.2, 17.0, 17.0, 16.9, 13.4, 13.2, 12.9, 12.7;

HRMS (ESI): calc. for $C_{17}H_{34}O_6Si_2Na$ $[M+Na]^+$: 413.1786, found: 413.1782.

Synthesis of diol 7:

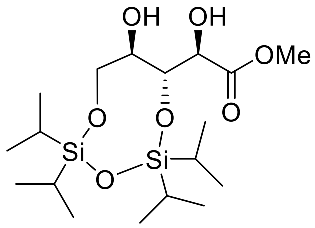

$C_{18}H_{38}O_7Si_2$
422.67 g/mol
7

The TIPDSi-protected alcohol **6** (6.18 g, 15.83 mmol, 1.00 equiv) was dissolved in dry methanol (122 mL). After the addition of Bu_2SnO (400 mg, 1.58 mmol, 0.10 equiv), the stirred white suspension was heated to 65 °C for 3 h before cooled to room temperature. The reaction mixture was quenched by the addition of an aqueous $NaHCO_3$ solution (80 mL) and EtOAc (200 mL) was added. The layers were separated and the aqueous layer was extracted with EtOAc (3 × 150 mL). After filtration of the combined organic layers through celite®, the filtrate was dried over Na_2SO_4 and concentrated in vacuo. The crude product was purified by flash column chromatography (PE/EtOAc 5:1 → 3:1) delivering diol **7** (4.79 g, 11.3 mmol, 72%) as yellow oil.

R_f (PE:EtOAc = 2:1) = 0.34;

$[\alpha]_D^{20}$ = −17.7 (*c* = 1.0, $CHCl_3$);

^{1}H NMR (400 MHz, $CDCl_3$): δ [ppm] = 4.53 (bs, 1 H), 4.15–4.09 (m, 2 H), 4.06 (dd, *J* = 9.6, 1.5 Hz, 1 H), 3.87 (d, *J* = 9.4 Hz, 1 H), 3.82 (dd, *J* = 11.7, 2.1 Hz, 1 H), 3.77 (s, 3 H), 3.10 (bs, 1 H), 1.09–1.02 (m, 28 H)

^{13}C NMR (100 MHz, $CDCl_3$): δ [ppm] = 173.2, 73.8, 73.0, 70.6, 62.4, 52.6, 17.5, 17.5, 17.4, 17.4, 17.4, 17.4, 17.4, 17.4, 17.3, 13.5, 13.5, 12.7, 12.7;

HRMS (ESI): calc. for $C_{18}H_{38}O_7Si_2Na$ $[M+Na]^+$: 445.2048, found: 445.2044.

Synthesis of dimethylated TIPDS-protected ether 8:

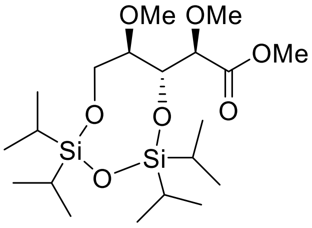

$C_{20}H_{42}O_7Si_2$

450.72 g/mol

8

Trimethyloxonium tetrafluoroborate (13.4 g, 90.6 mmol, 8.00 equiv) was washed with CH_2Cl_2 (3 × 50 mL) under an argon atmosphere and dried under high vacuum. Subsequently, the dried trimethyloxonium tetrafluoroborate and proton sponge® (18.7 g, 87.1 mmol, 7.70 equiv) were dissolved in dry CH_2Cl_2 (566 mL). The TIPDSi-protected diol **7** (4.79 g, 11.3 mmol, 1.00 equiv) was dissolved in dry CH_2Cl_2 (15.0 mL) and added dropwise to the pale yellow suspension. The orange reaction mixture was stirred at room temperature for 28 h. After the addition of an aqueous $NaHCO_3$ solution (300 mL) and dilution with CH_2Cl_2 (500 mL), the layers were separated. The combined organic layer was washed with an aqueous $KHSO_4$ solution (1 M, 3 × 500 mL) and brine (500 mL), dried over Na_2SO_4 and the solution was concentrated in vacuo. The crude product was purified by flash column chromatography (PE/EtOAc 4:1) delivering the dimethylated TIPDSi-protected ether **8** (4.46 g, 9.88 mmol, 88%) as yellow oil.

$\mathbf{R_{\mathit{f}}}$ **(**PE:EtOAc = 2:1) = 0.73;

$[\alpha]_D^{20}$ = −28.77 (*c* = 1.0, $CHCl_3$);

^{1}H NMR (400 MHz, $CDCl_3$): δ [ppm] = 4.35 (dd, *J* = 9.7, 1.5 Hz, 1 H), 3.99 (dd, *J* = 8.8, 1.4 Hz, 2 H), 3.89 (dd, *J* = 12.3, 1.4 Hz, 1 H), 3.69 (s, 3 H), 3.51 (s, 3 H), 3.36 - 3.33 (m, 4 H), 1.28–1.01 (m, 28 H);

^{13}C NMR (100 MHz, $CDCl_3$): δ [ppm] = 170.4, 82.7, 80.2, 71.3, 59.8, 58.8, 57.7, 51.7, 17.6, 17.5, 17.5, 17.4, 17.4, 17.4, 17.3, 17.2, 13.5, 13.5, 12.7, 12.5;

HRMS (ESI): calc. for $C_{20}H_{42}O_7Si_2Na$ $[M+Na]^+$: 473.2361, found: 473.2366.

Synthesis of aliphatic diol 9:

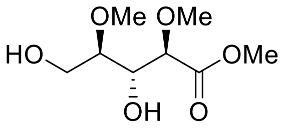

$C_8H_{16}O_6$
208.21 g/mol
9

The dimethylated TIPDSi-protected ether **8** (5.31 g, 11.8 mmol, 1.00 equiv) was dissolved in dry THF (59 mL). Afterwards, Et_3N (2.5 mL, 17 mmol, 1.50 equiv) and $HF{\cdot}Et_3N$ (5.8 mL, 36 mmol, 3.00 equiv) were added in one portion, respectively. The reaction mixture was stirred at room temperature for 3 h before SiO_2 (14.2 g, 235 mmol, 20.00 equiv) was added through a dropping funnel. After no further gas formation was observed, the reaction mixture was concentrated in vacuo. The crude product was filtered through a short flash column (PE/EtOAc 1:9) providing the crude aliphatic diol **9** (2.04 g, 9.79 mmol, 83%) as colorless oil.

R_f (PE:EtOAc = 1:5) = 0.15;

$[\alpha]_D^{20}$ = +15.7 (*c* = 1.0, CH_3OH);

^{1}H NMR (700 MHz, CD_3OD): δ [ppm] = 3.99 (dd, *J* = 8.6, 3.2 Hz, 1 H), 3.95 (d, *J* = 3.4 Hz, 1 H), 3.84 (dd, *J* = 11.9, 2.9 Hz, 1 H), 3.71 (s, 3 H), 3.62 (dd, *J* = 12.0, 4.3 Hz, 1 H), 3.45 (s, 3 H), 3.33 (s, 3 H), 3.32–3.31 (m, 1 H);

^{13}C NMR (175 MHz, CD_3OD): δ [ppm] = 172.6, 83.5, 81.7, 72.4, 61.2, 59.6, 58.3, 52.1;

HRMS (ESI): calc. for $C_8H_{16}O_6H$ $[M+H]^+$: 209.1020, found: 209.1016.

Synthesis of TBD-diprotected ether 10:

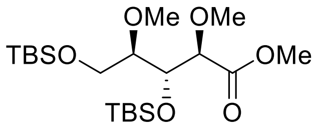

$C_{20}H_{44}O_6Si_2$
436.74 g/mol
10

The crude diol **9** (2.04 g, 9.79 mmol, 1.00 equiv) was dissolved in dry CH_2Cl_2 (21 mL) and cooled to −78 °C. To this solution, 2,6-lutidine (6.8 mL, 58 mmol, 6.00 equiv) was added dropwise, followed by the dropwise addition of TBSOTf (6.7 mL, 29 mmol, 3.00 equiv). The reaction mixture was further stirred for 5 min at −78 °C, then warmed to 0 °C and stirred for 3 h at this temperature. Then, an aqueous $NaHCO_3$ solution (15 mL) was added and the mixture warmed to room temperature. The layers were separated and the aqueous layer was extracted with CH_2Cl_2 (3 × 30 mL). The combined organic layers were washed with water (50 mL) and an aqueous $KHSO_4$ solution (1 M, 50 mL), dried over Na_2SO_4 and concentrated in vacuo. The crude product was purified by flash column chromatography (PE/EtOAc 4:1) giving the TBS-diprotected ether **10** (4.08 g, 9.34 mmol, 95%) as colorless oil.

$\mathbf{R_{\mathit{f}}}$ **(**PE:EtOAc = 10:1) = 0.40;

$[\alpha]_D^{20}$ = +5.2 (*c* = 1.0, $CHCl_3$);

^{1}H NMR (400 MHz, $CDCl_3$): δ [ppm] = 4.06 (dd, *J* = 7.0, 3.9 Hz, 1 H), 3.91 (d, *J* = 3.9 Hz, 1 H), 3.84 (dd, *J* = 11.0, 3.1 Hz, 1 H), 3.71 (s, 3H), 3.63 (dd, *J* = 11.0, 5.5 Hz, 1 H), 3.44 (s, 3H), 3.39–3.35 (m, 4H), 0.89 (s, 9 H), 0.87 (s, 9 H), 0.09 (s, 3 H), 0.08 (s, 3 H), 0.04 (s, 6 H);

^{13}C NMR (100 MHz, $CDCl_3$): δ [ppm] = 171.0, 82.5, 81.7, 73.1, 92.8, 59.1, 58.7, 51.7, 26.1, 26.1, 26.1, 25.9, 25.9, 25.9, 18.5, 18.1, −4.6, −4.6, −5.1, −5.3;

HRMS (ESI): calc. for $C_{20}H_{44}O6_7Si_2Na$ $[M+Na]^+$: 459.2574, found: 459.2574.

Synthesis of TBS-protected ether 11:

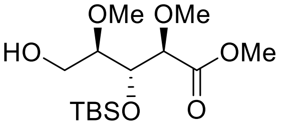

$C_{14}H_{30}O_6Si$
322.47 g/mol
11

Pyridinium *p*-toluenesulfonate (130 mg, 1.22 mmol, 1.30 equiv) was added to a solution of the TBS-diprotected ether **10** (410 mg, 0.94 mmol, 1.00 equiv) in methanol (9.4 mL). The reaction mixture was stirred at room temperature for 19 h before an aqueous $NaHCO_3$ solution (8.0 mL) was added and stirred further for 40 min. The layers were separated, the aqueous layer extracted with EtOAc (2 × 20 mL), the combined organic layer dried over Na_2SO_4 and concentrated in vacuo. The crude product was purified by flash column chromatography (PE/EtOAc 2:1) providing the TBS-protected ether **11** (287 mg, 0.89 mmol, 95%) as colorless oil.

R_f (PE:EtOAc = 4:1) = 0.21;

$[\alpha]_D^{20}$ = +4.8 (*c* = 0.23, $CHCl_3$);

^{1}H NMR (400 MHz, $CDCl_3$): δ [ppm] = 4.14 (dd, *J* = 7.1, 3.6 Hz, 1 H), 3.89 (d, *J* = 3.7 Hz, 1 H), 3.78 (dd, *J* = 11.8, 3.7 Hz, 1 H), 3.73 (s, 3 H), 3.66 (dd, *J* = 11.8, 3.7 Hz, 1 H), 3.46 (s, 3 H), 3.47–3.41 (m, 1 H), 3.36 (s, 3 H), 0.88 (s, 9 H), 0.11 (s, 3 H), 0.10 (s, 3 H);

^{13}C NMR (100 MHz, $CDCl_3$): δ [ppm] = 170.8, 82.5, 80.6, 72.9, 60.0, 59.3, 57.9, 51.8, 25.9, 25.9, 25.9, 18.1, −4.7, −4.8;

HRMS (ESI): calc. for $C_{14}H_{30}O_6SiNa$ $[M+Na]^+$: 345.1709, found: 345.1709.

Synthesis of amide 3:

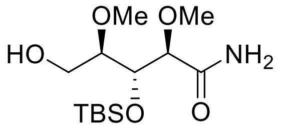

$C_{13}H_{29}NO_5Si$
307.46 g/mol
3

For this reaction no dry glassware is necessary. The TBS-protected ether **11** (42.0 mg, 0.13 mmol, 1.00 equiv) was dissolved in NH_3·MeOH (7 M, 869 µL) and stirred at room temperature for 27 h. The reaction mixture was concentrated in vacuo before flash column chromatography (PE/EtOAc 1:1) provided the amide **3** (41.0 mg, 0.13 mmol, >99%) as pale yellow solid.

$\mathbf{R_f}$ **(**PE:EtOAc = 1:6) = 0.15;

$[\alpha]_D^{20}$ = +12.7 (*c* = 0.65, MeOH);

^{1}H NMR (400 MHz, $CDCl_3$): δ [ppm] = 6.49 (bs, 1 H), 5.88 (bs, 1 H), 4.29 (dd, *J* = 7.3, 2.2 Hz, 1 H), 3.82 (dd, *J* = 11.8, 3.8 Hz, 1 H), 3.81(d, *J* = 2.2 Hz, 1 H), 3.63 (dd, *J* = 11.8, 3.5 Hz, 1 H), 3.51(s, 3 H), 3.42–3.38 (m, 1 H), 3.07 (s, 3 H), 2.22 (bs, 1 H), 0.88 (s, 9 H), 0.13 (s, 3 H), 0.12 (s, 3 H);

^{13}C NMR (125 MHz, $CDCl_3$): δ [ppm] = 173.1, 84.3, 80.8, 72.5, 59.9, 59.6, 57.4, 26.0, 26.0, 26.0, 18.2, −4.2, −5.1;

HRMS (ESI): calc. for $C_{13}H_{29}NO_5Si_2Na$ $[M+Na]^+$: 330.1707, found: 330.1704.

Synthesis of 3-chloro-4-hydroxybenzaldehyde 13:

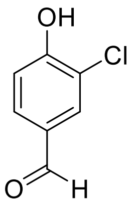

$C_7H_5ClO_2$
156.57 g/mol
13

To a solution of 4-hydroxybenzaldehyde (**12**, 500 mg, 4.09 mmol, 1.00 equiv) in dry $CHCl_3$ (20 mL) was added in one portion NCS (0.55 g, 4.09 mmol, 1.00 equiv). The reaction mixture was stirred under exclusion of light at 50 °C for 23 h. After cooling to room temperature, the reaction mixture was concentrated and the residue dissolved in CH_2Cl_2 (20 mL). The organic layer was washed with water (30 mL) and dried over Na_2SO_4. After removal of the solvent, 3-chloro-4-hydroxybenzaldehyde (**13**, 0.51 g, 3.73 mmol, 91%) was obtained as yellow oil.

P. K. S. Sarma, V. P. Acharya, S. R. Kasibhatla, V. N. Viswanadhan, P. Shekhar, A. Bischoff, US 2010/0280067.

The obtained 3-chloro-4-hydroxybenzaldehyde (**13**) was used in the next step without detailed characterization.

Synthesis of TBS-protected benzylic aldehyde 14:

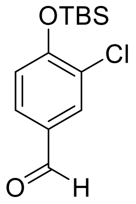

$C_{13}H_{19}ClO_2Si$
270.83 g/mol

14

3-Chloro-4-hydroxybenzaldehyde (**13**, 250 mg, 1.60 mmol, 1.00 equiv) was dissolved in dry CH_2Cl_2 (3.4 mL) and cooled to −78 °C. 2,6-Lutidine (0.6 mL, 4.8 mmol, 3.00 equiv) was added dropwise to the suspension, followed by the dropwise addition of TBSOTf (0.5 mL, 2.4 mmol, 1.50 equiv). The reaction mixture was warmed to 0 °C over a period of 30 min, stirred at this temperature for further 4 h before the addition of an aqueous $NaHCO_3$ solution (5.0 mL) and warming to room temperature. The layers were separated and the aqueous layer was extracted with CH_2Cl_2 (3 × 5.0 mL), the combined organic layer was washed with an aqueous $KHSO_4$ solution (1 M, 10.0 mL), brine (10.0 mL) and dried over Na_2SO_4. The organic layer was concentrated in vacuo. The crude product was purified by flash column chromatography (PE/EtOAc 20:1) providing the TBS-protected benzylic aldehyde **14** (0.26 g, 0.98 mmol, 61%) as yellow oil.

R_f (PE:EtOAc = 20:1) = 0.41;

^{1}H NMR (400 MHz, $CDCl_3$): δ [ppm] = 9.85 (s, 1 H), 7.90 (d, *J* = 2.0 Hz, 1 H), 7.67 (dd, *J* = 8.2, 2.6 Hz, 1 H), 6.99 (d, *J* = 8.6 Hz, 1 H), 1.04 (s, 9 H), 0.28 (s, 6 H);

^{13}C NMR (100 MHz, $CDCl_3$): δ [ppm] = 189.9, 157.3, 132.0, 131.1, 129.8, 127.0, 120.7, 25.7 (3C), 18.5, −4.5 (2C);

J. Boukouvalas, F. Maltais, N. Lachance, *Tetrahedron Lett.* **1994**, *35*, 7897–7900.

HRMS (ESI): calculated for $C_{13}H_{19}ClO_2SiH$ $[M+H]^+$: 271.0916, found:271.0912.

Synthesis of dibromide 15:

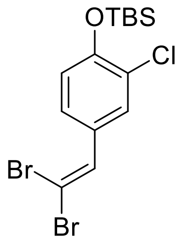

$C_{14}H_{19}Br_2ClOSi$
426.65 g/mol
15

CBr_4 (0.68 g, 2.0 mmol, 1.10 equiv) was dissolved in dry CH_2Cl_2 (18.5 mL) and cooled to 0 °C. After the addition of PPh_3 (1.07 g, 4.07 mmol, 2.20 equiv), the yellow reaction mixture was stirred at the same temperature for 15 min before the dropwise addition of TBS-protected benzylic aldehyde **14** (500 mg, 1.85 mmol, 1.00 equiv). After 5 h, the reaction was quenched with an aqueous NH_4Cl solution (20.0 mL) at 0 °C. The layers were separated and the aqueous layer was extracted with CH_2Cl_2 (3 × 20.0 mL). The combined organic layer was dried over Na_2SO_4 and concentrated in vacuo. The crude product was purified by flash column chromatography (PE/EtOAc = 50:1) providing dibromide **15** (0.75 g, 1.8 mmol, 95%) as yellow oil.

R_f (PE:EtOAc = 50:1) = 0.83;

^{1}H NMR (400 MHz, $CDCl_3$): δ [ppm] = 7.95 (d, *J* = 2.4 Hz, 1 H), 7.34 (s, 1 H), 7.32 (dd, *J* = 8.8, 2.7 Hz, 1 H), 6.86 (d, *J* = 8.5 Hz, 1 H), 1.04 (s, 9 H), 0.25 (s, 6 H);

^{13}C NMR (100 MHz, $CDCl_3$): δ [ppm] = 151.9, 135.3, 130.5, 129.4, 128.0, 125.8, 120.5, 89.0, 25.7 (3C), 18.5, −4.5 (2C);

HRMS (ESI): not found.

Synthesis of (*Z*)-bromide 4:

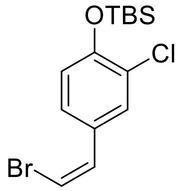

$C_{14}H_{20}BrClOSi$
347.75 g/mol

4

$Pd(OAc)_2$ (8.0 mg, 30 µmol, 1 mol %) and PPh_3 (35.0 mg, 14 µmol, 0.16 equiv) were dissolved in toluene (8.0 mL) and stirred at room temperature for 15 min. Then, dibromide **15** (0.36 g, 0.84 mmol, 1.00 equiv) as well as Bu_3SnH (0.20mL , 0.85 mmol, 1.00 equiv) were added dropwise to the solution before stirring at room temperature for 1 h. The reaction mixture was diluted with petroleum ether (4.0 mL) and the layers were separated. The organic layer was washed with water (3 × 4.0 mL), dried over Na_2SO_4 and concentrated in vacuo. The crude product was purified by flash column chromatography (PE) providing (*Z*)-bromide **4** (0.21 g, 0.63 mmol, 74%) as yellow oil.

R_f **(**PE:EtOAc = 99:1) = 0.86;

^{1}H NMR (400 MHz, $CDCl_3$): δ [ppm] = 7.74 (d, *J* = 2.4 Hz, 1 H), 7.50 (dd, *J* = 8.5, 2.4 Hz, 1 H), 6.93 (d, *J* = 8.2 Hz, 1 H), 6.88 (d, *J* = 8.5 Hz, 1 H), 6.35 (d, *J* = 8.2 Hz, 1 H), 1.04 (s, 9 H), 0.25 (s, 6 H);

^{13}C NMR (100 MHz, $CDCl_3$): δ [ppm] = 151.7, 131.0, 130.8, 129.2, 128.6, 125.5, 120.4, 105.8, 25.8 (3C), 18.5, −4.2 (2C);

HRMS (ESI): calculated for $C_{14}H_{20}ClO_2SiH$ $[M+H]^+$: 347.0228, found:347.0224.

General procedure for the Buchwald-type reaction:

All reactions were conducted in flasks without drying them before. The amide (1.00 equiv) and K_2CO_3 (1.30 equiv) were propounded and the flask was evacuated and flushed with argon three times. Then, THF (0.06 M) and the (*Z*)-bromide (1.00 equiv) were added. A stock solution of the Cu source (0.30 equiv) and the ligand (0.60 equiv) was prepared in dry THF (0.12 M) before the addition of the required portion to the reaction mixture. The reaction mixture was stirred at 65 °C for 19–22 h before cooling to room temperature and quenching by the addition of an aqueous NH_4Cl solution. The layers were separated and the organic layer was washed with water, brine, dried over Na_2SO_4, filtration and concentrated in vacuo. The crude product was purified by flash column chromatography providing the Buchwald product given below.

Cu-source: CuI (after purchase sublimated); ligand: DMEDA, stirring time: 19 h, 54% of **16**

scale: 48 μmol (amide)

Flash column chromatography (PE:EtOAc = 9:1 → 4:1 → 2:1)

R*f* (PE:EtOAc = 2:1) = 0.47;

[α]$_D^{20}$ = +17.7 (*c* = 1.7, CH_2Cl_2);

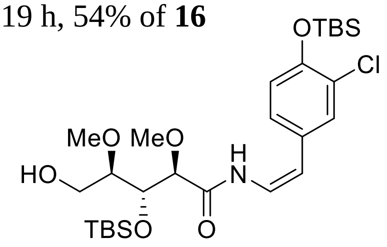

$C_{27}H_{48}ClNO_6Si_2$
574.30 g/mol

16

^{1}H NMR (400 MHz, $CDCl_3$): δ [ppm] = 8.87 (d, *J* = 11.6 Hz, 1 H), 7.30 (d, *J* = 2.0 Hz, 1 H), 7.10 (dd, *J* = 8.4, 2.2 Hz, 1 H), 6.88 (d, *J* = 8.5 Hz, 1 H), 6.87 (dd, *J* = 11.6, 9.6 Hz, 1 H), 5.66 (d, *J* = 9.6 Hz, 1H), 4.22 (dd, *J* = 8.2, 1.7 Hz, 1 H), 3.94 (d, *J* = 1.7 Hz, 1H), 3.81 (dd, *J* = 11.8, 3.2 Hz, 1 H), 3.65–3.58 (m, 1 H), 3.57 (s, 3 H), 3.43–3.39 (m, 1 H), 3.33 (s, 3 H), 1.03 (s, 9 H), 0.81 (s, 9 H), 0.24 (s, 6 H), 0.10 (s, 3 H), 0.04 (s, 3 H);

^{13}C NMR (125 MHz, $CDCl_3$): δ [ppm] = 167.9, 150.5, 130.0, 129.5, 127.4, 126.2, 121.4, 121.0, 109.3, 84.1, 80.4, 72.4, 60.0, 59.0, 57.4, 25.9, 25.9, 25.9, 25.8, 25.8, 25.8, 18.5, 18.1, −4.2, −4.2, −4.2, −5.1,

HRMS (ESI): calculated for $C_{27}H_{48}ClNO_6Si_2Na$ $[M+Na]^+$: 596.2601, found: 596.2602 .

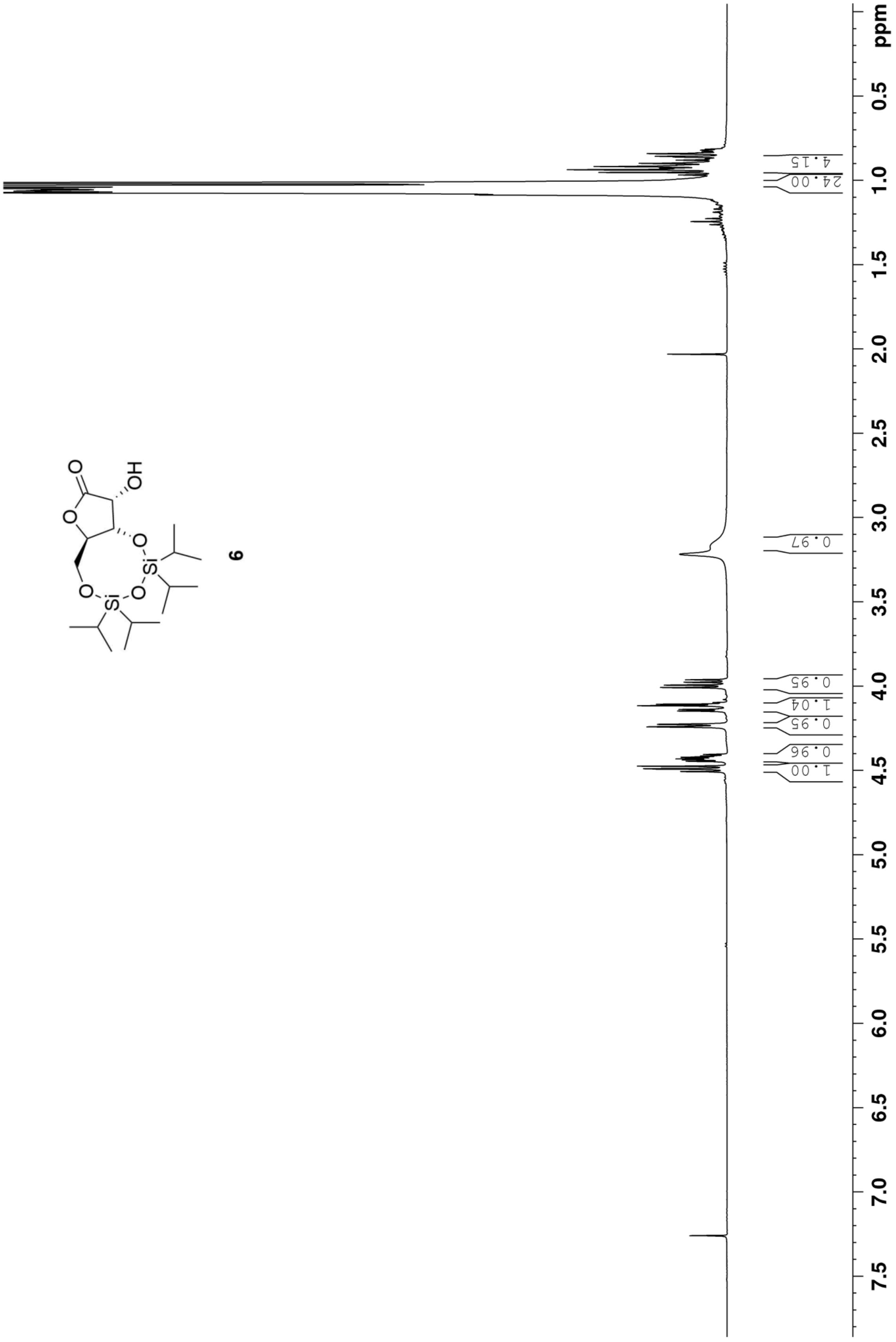
6
ppm
0.5
1.0
1.5
2.0
2.5
3.0
3.5
4.0
4.5
5.0
5.5
6.0
6.5
7.0
7.5
4.15
24.00
0.97
0.95
1.04
0.95
0.96
1.00

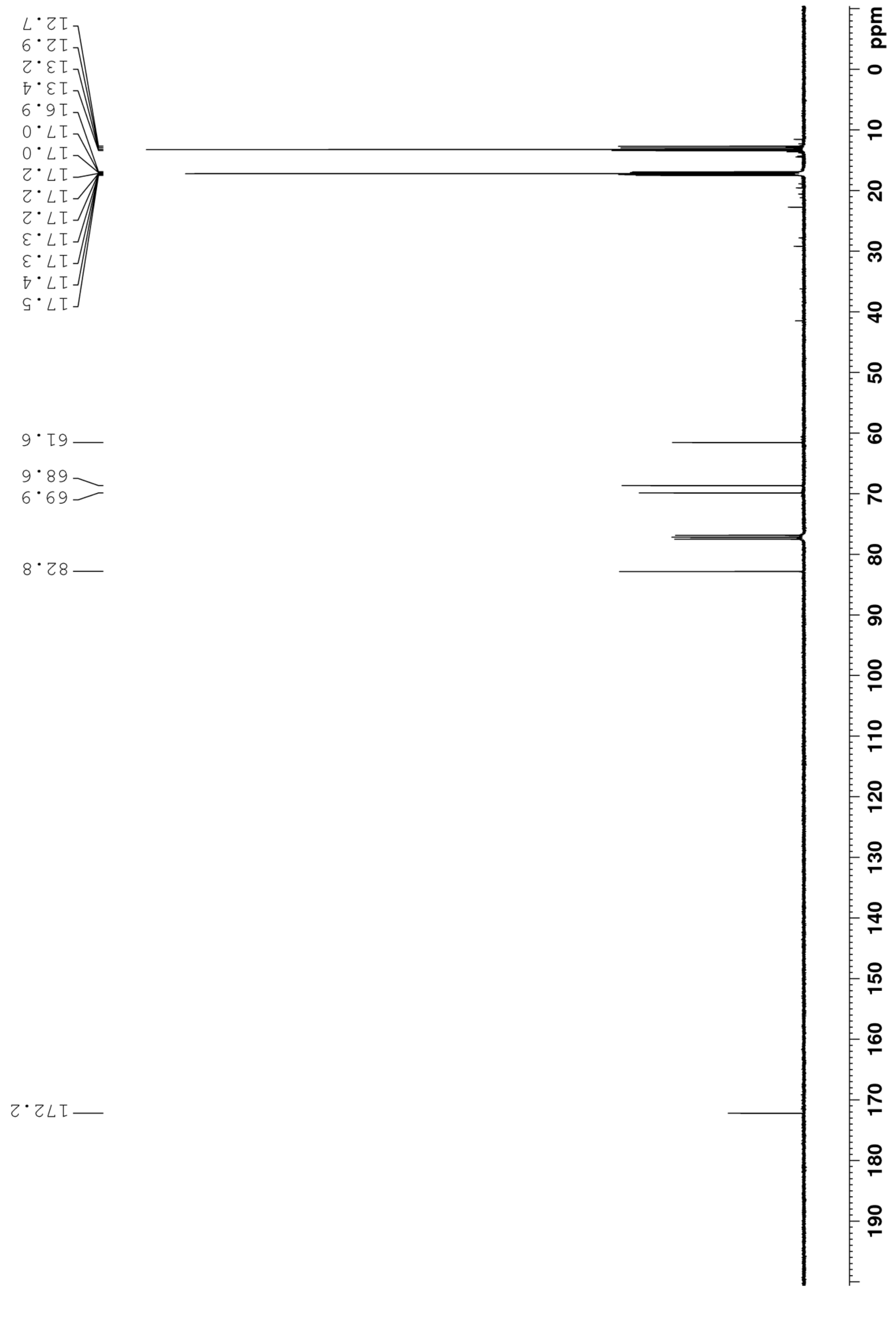
12.7
12.9
13.2
13.4
16.9
17.0
17.0
17.2
17.2
17.2
17.3
17.3
17.4
17.5
61.6
68.6
69.9
82.8
172.2
ppm
0
10
20
30
40
50
60
70
80
90
100
110
120
130
140
150
160
170
180
190

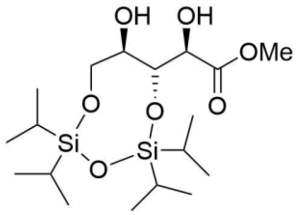
OH
OH
OMe
O
O
O
Si
O
Si

7

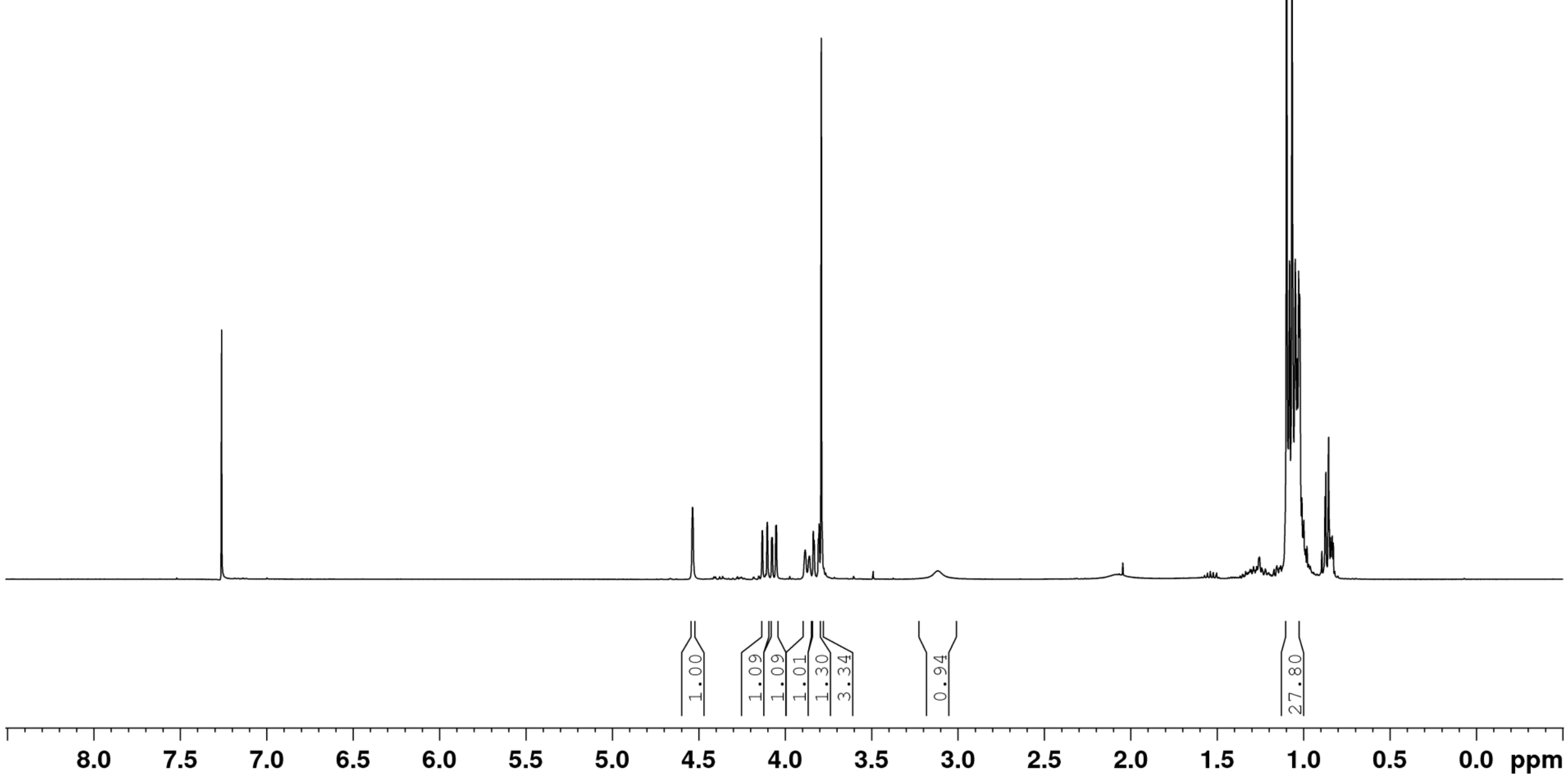
1.00
1.09
1.09
1.01
1.30
3.34
0.94
27.80
8.0
7.5
7.0
6.5
6.0
5.5
5.0
4.5
4.0
3.5
3.0
2.5
2.0
1.5
1.0
0.5
0.0
ppm

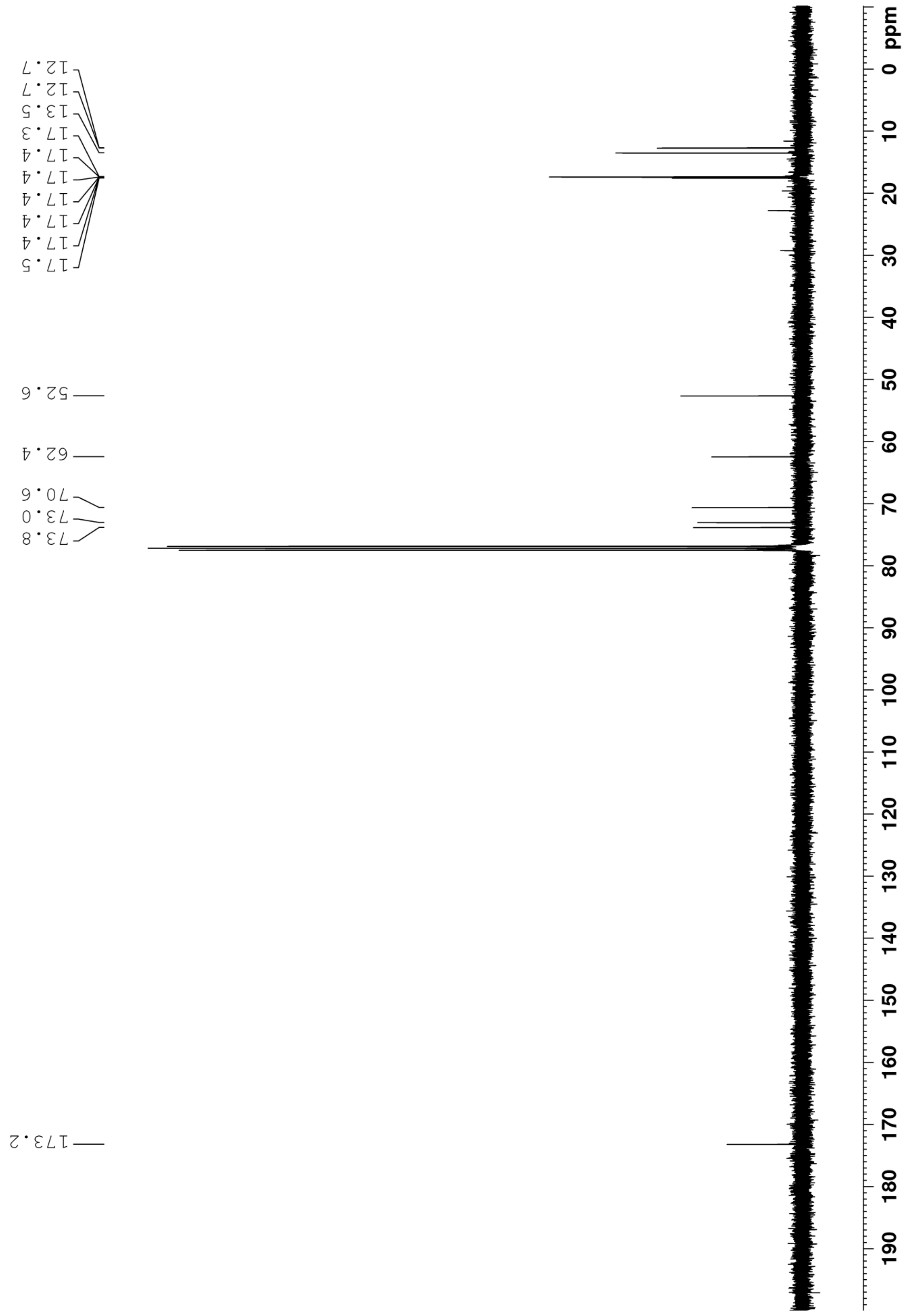
12.7
12.7
13.5
17.3
17.4
17.4
17.4
17.4
17.4
17.5
52.6
62.4
70.6
73.0
73.8
173.2
ppm

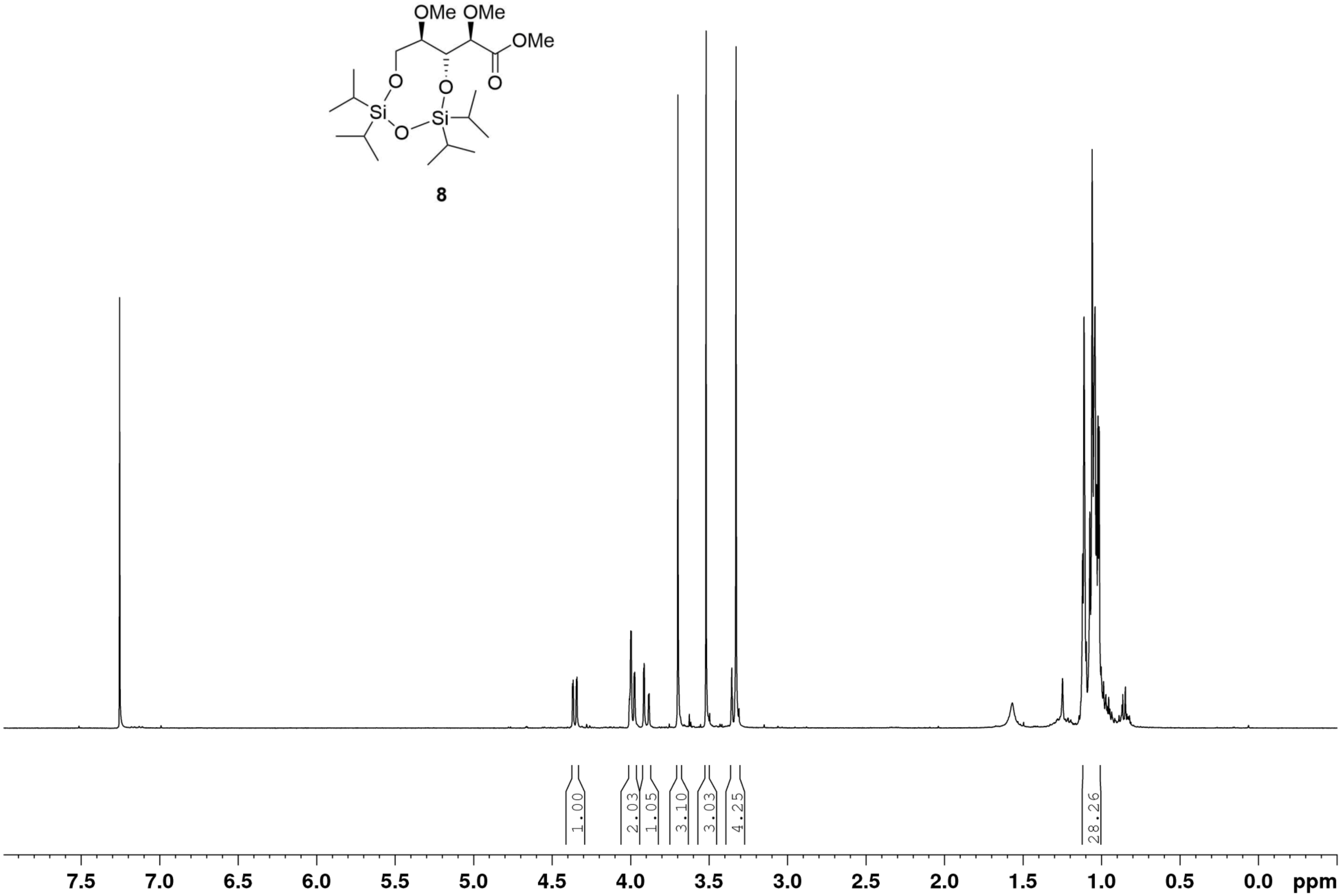
OMe OMe
OMe
O
O
O
Si
O
Si
8
1.00
2.03
1.05
3.10
3.03
4.25
28.26
7.5
7.0
6.5
6.0
5.5
5.0
4.5
4.0
3.5
3.0
2.5
2.0
1.5
1.0
0.5
0.0
ppm

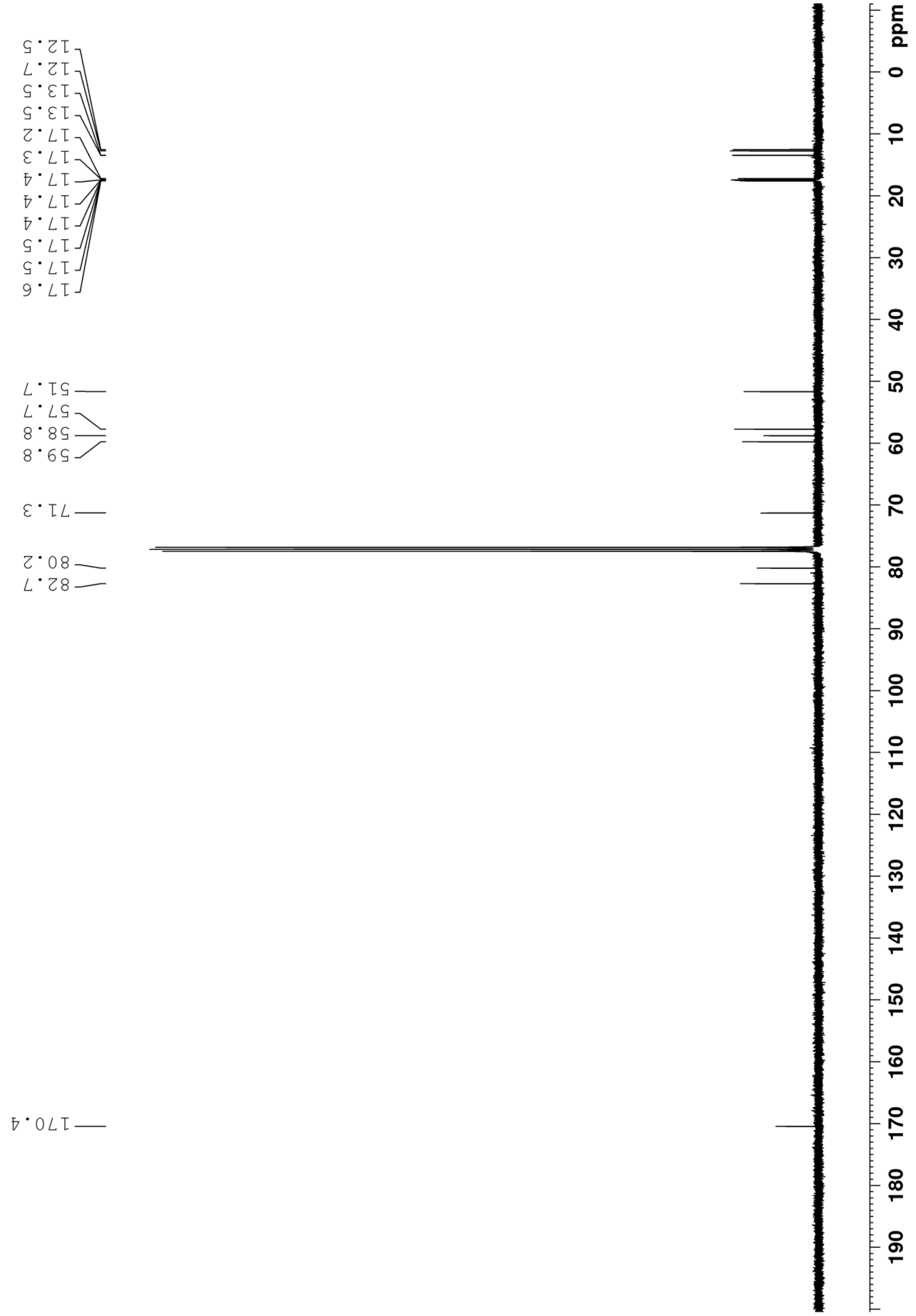
12.5
12.7
13.5
13.5
17.2
17.3
17.4
17.4
17.4
17.5
17.5
17.6
51.7
57.7
58.8
59.8
71.3
80.2
82.7
170.4
ppm

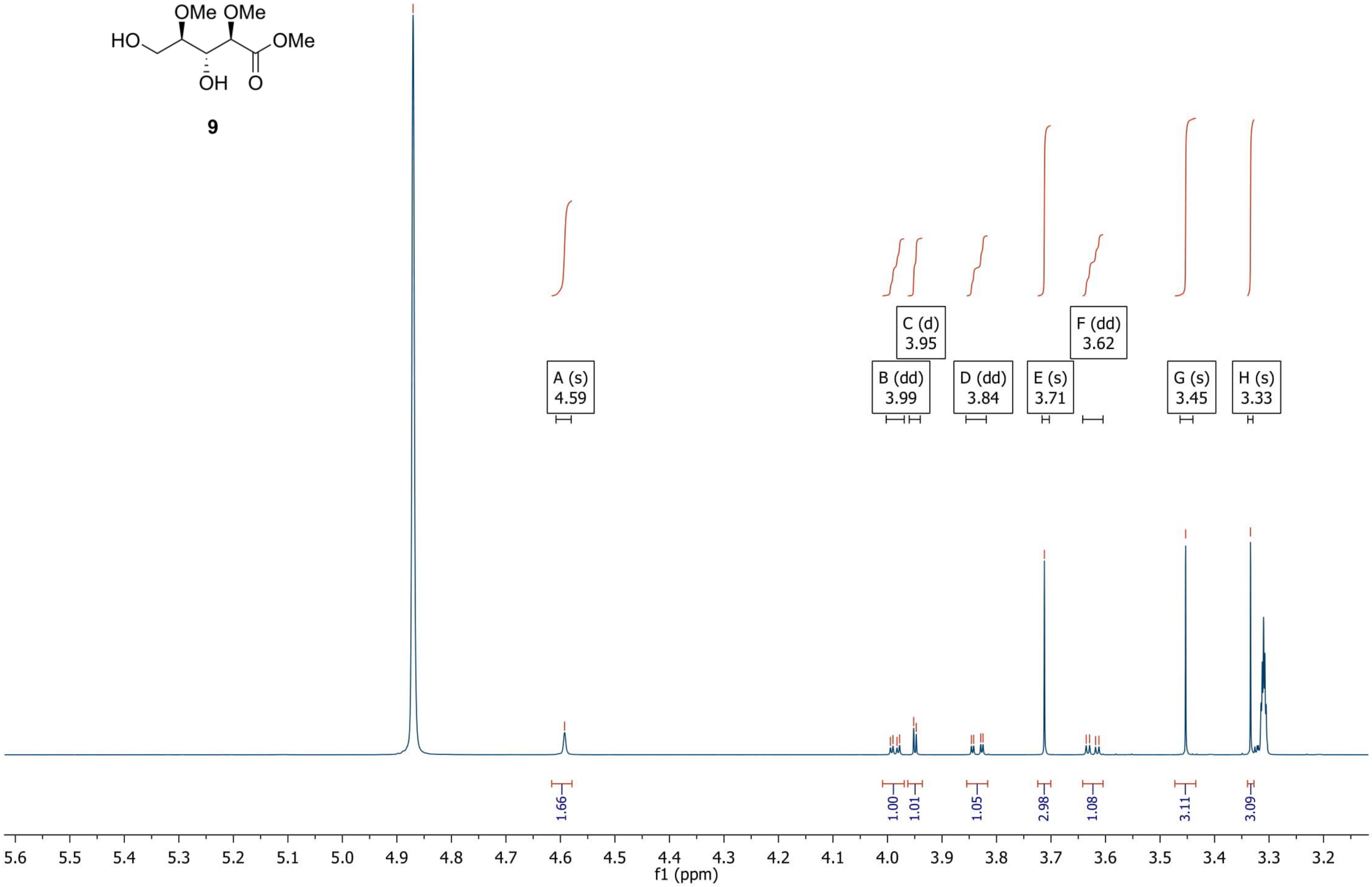
OMe OMe
HO
OMe
OH
O
A (s)
4.59
B (dd)
3.99
C (d)
3.95
D (dd)
3.84
E (s)
3.71
F (dd)
3.62
G (s)
3.45
H (s)
3.33
1.66
1.00
1.01
1.05
2.98
1.08
3.11
3.09
5.6 5.5 5.4 5.3 5.2 5.1 5.0 4.9 4.8 4.7 4.6 4.5 4.4 4.3 4.2 4.1 4.0 3.9 3.8 3.7 3.6 3.5 3.4 3.3 3.2
f1 (ppm)

9

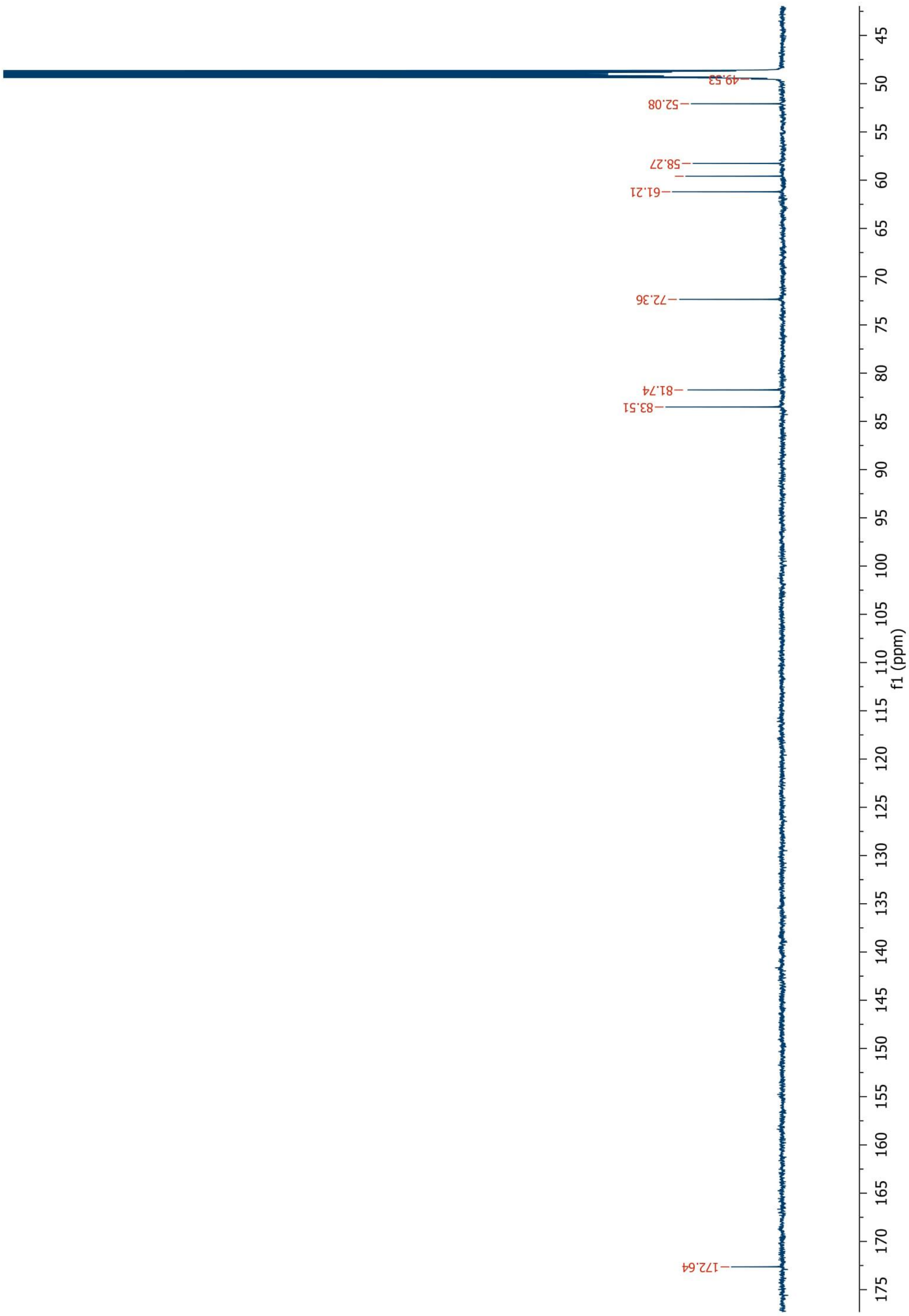
49.53
52.08
58.27
61.21
72.36
81.74
83.51
172.64
f1 (ppm)

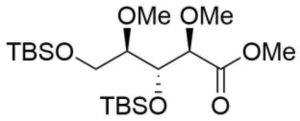

TBSO
OMe
OMe
OMe
TBSO
O

10

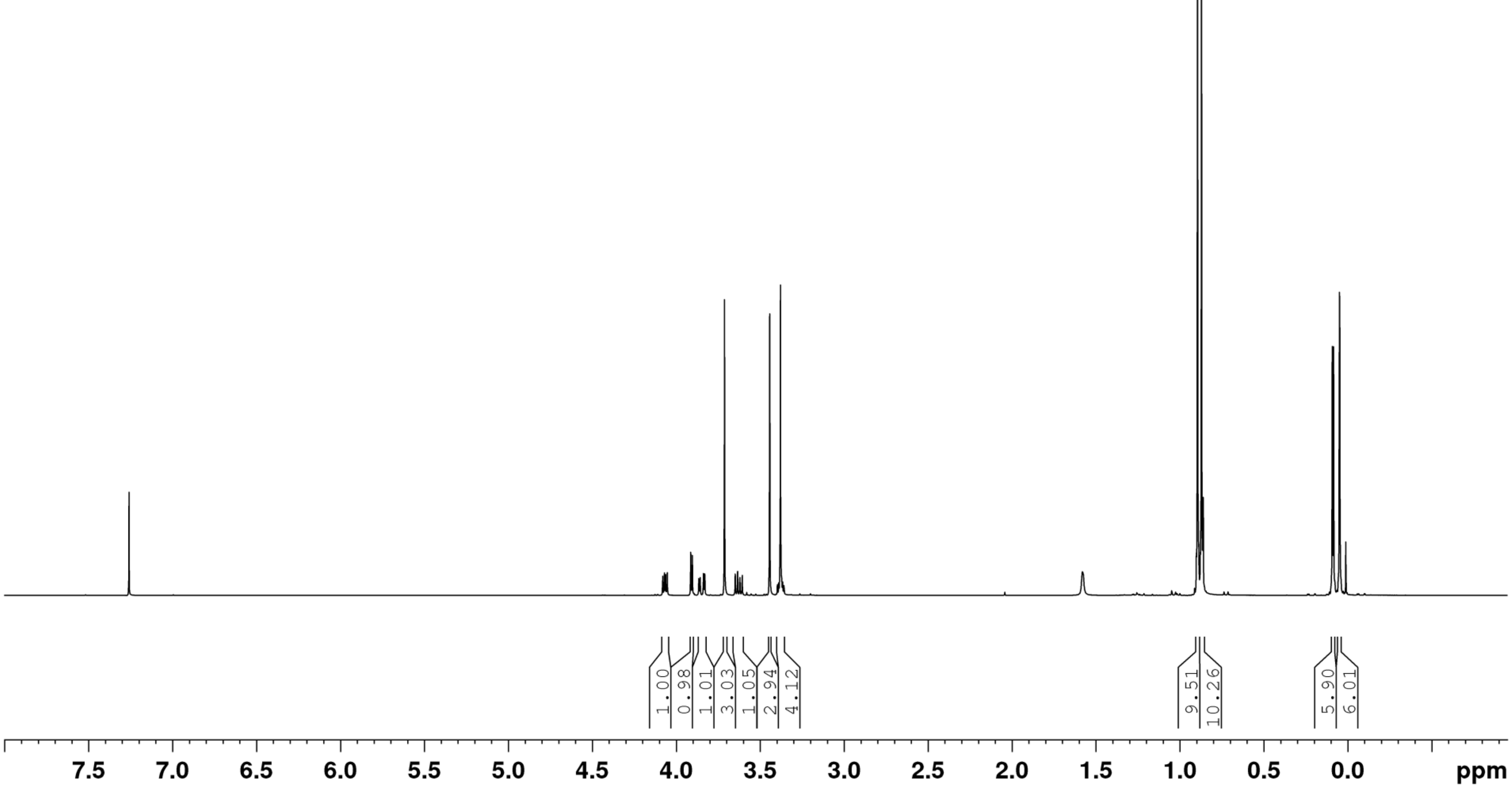

1.00
0.98
1.01
3.03
1.05
2.94
4.12
9.51
10.26
5.90
6.01
7.5
7.0
6.5
6.0
5.5
5.0
4.5
4.0
3.5
3.0
2.5
2.0
1.5
1.0
0.5
0.0
ppm

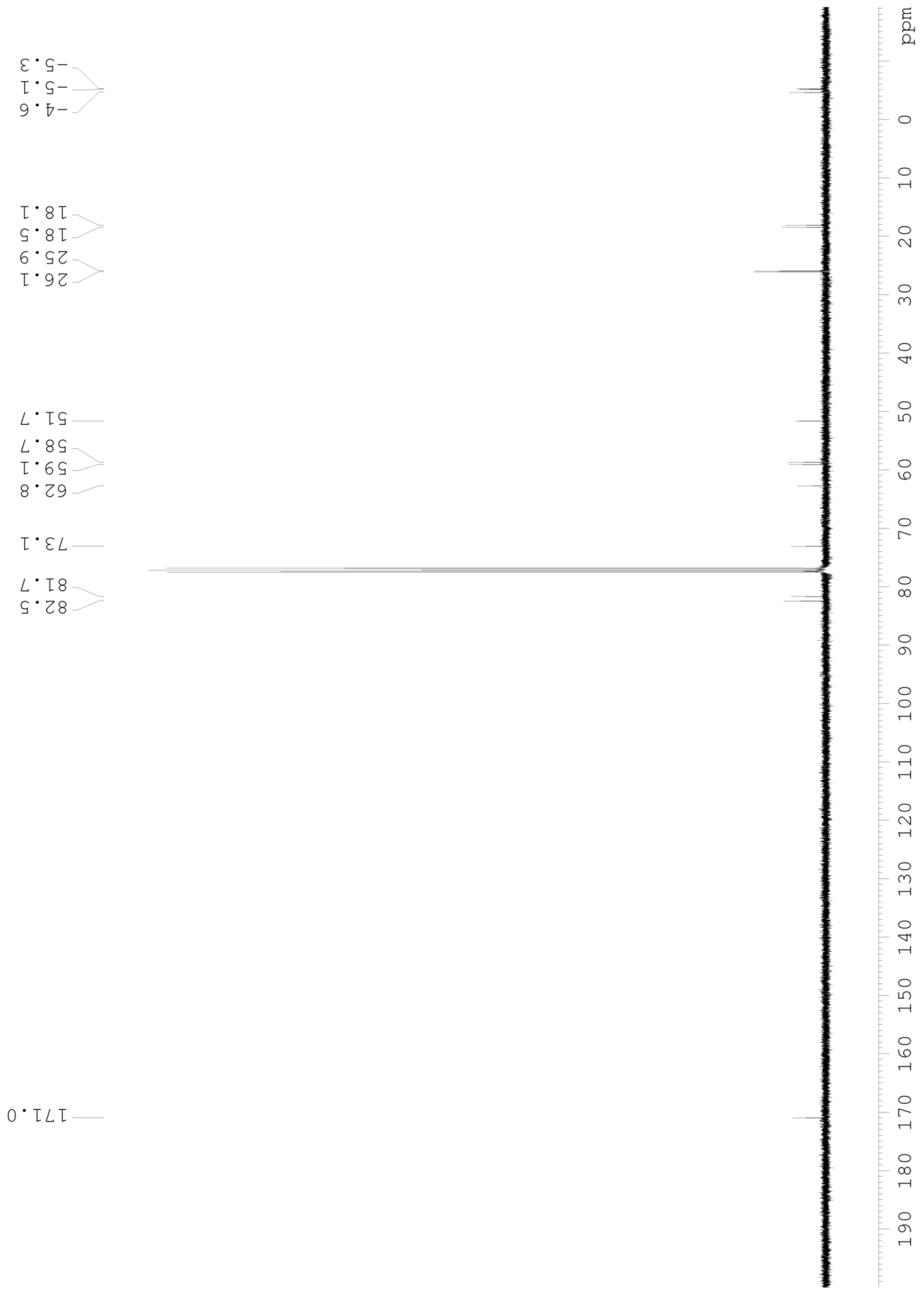

-5.3
-5.1
-4.6
18.1
18.5
25.9
26.1
51.7
58.7
59.1
62.8
73.1
81.7
82.5
171.0
ppm
0
10
20
30
40
50
60
70
80
90
100
110
120
130
140
150
160
170
180
190

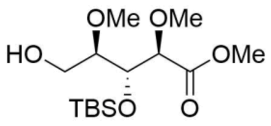

OMe OMe
HO
OMe
TBSO
O

11

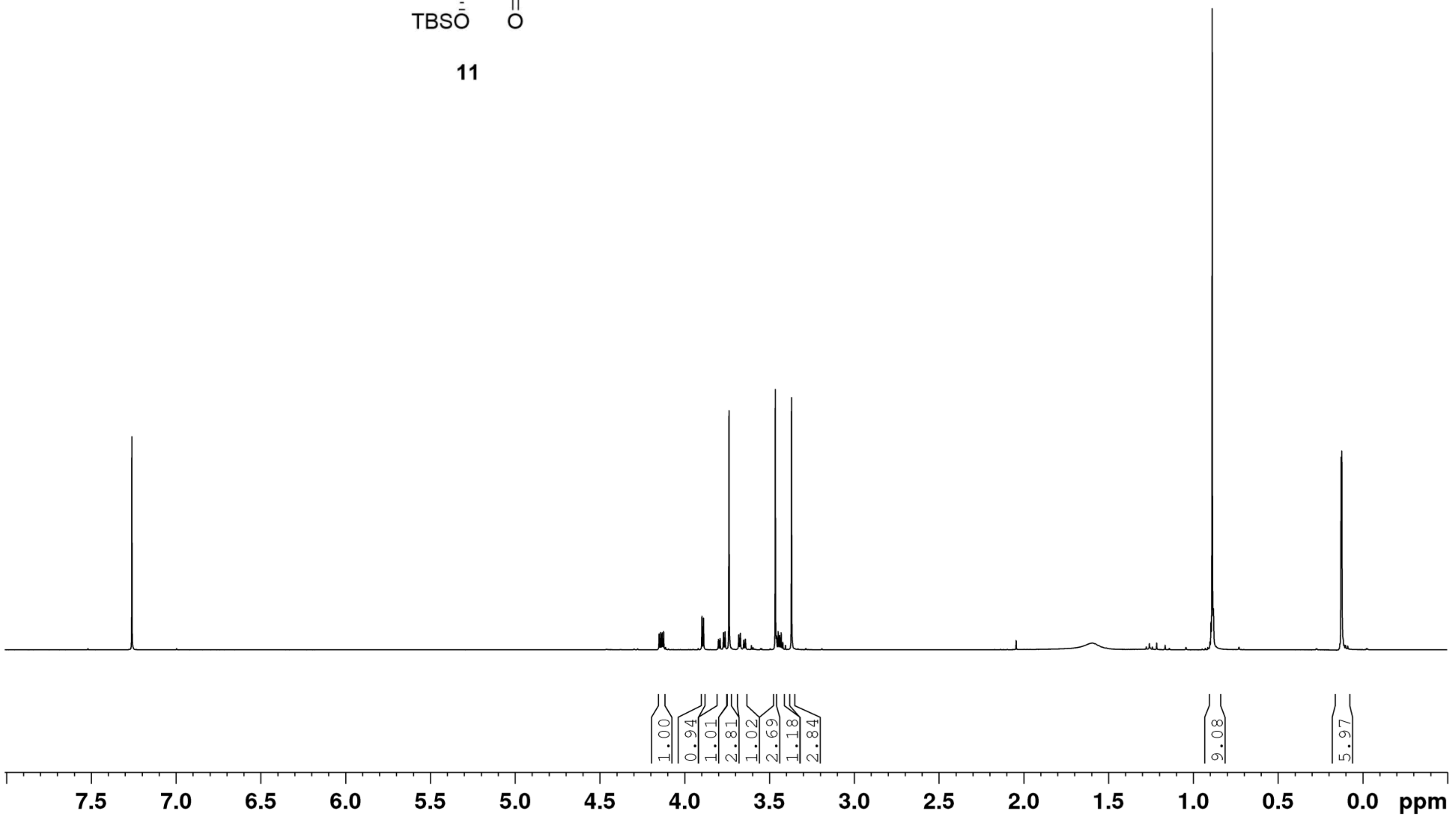

1.00
0.94
1.01
2.81
1.02
2.69
1.18
2.84
9.08
5.97
7.5
7.0
6.5
6.0
5.5
5.0
4.5
4.0
3.5
3.0
2.5
2.0
1.5
1.0
0.5
0.0
ppm

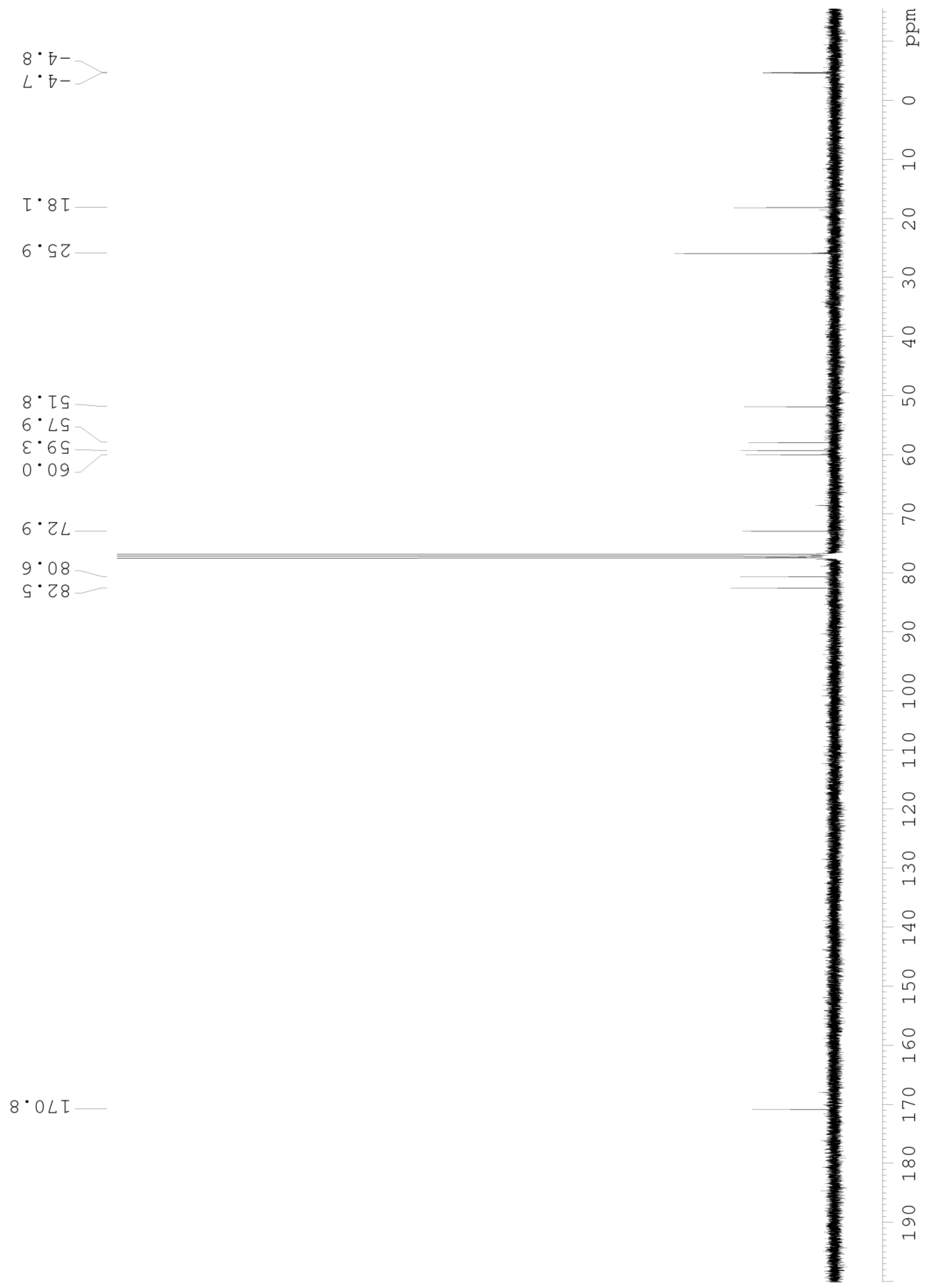
-4.8
-4.7
18.1
25.9
51.8
57.9
59.3
60.0
72.9
80.6
82.5
170.8
ppm

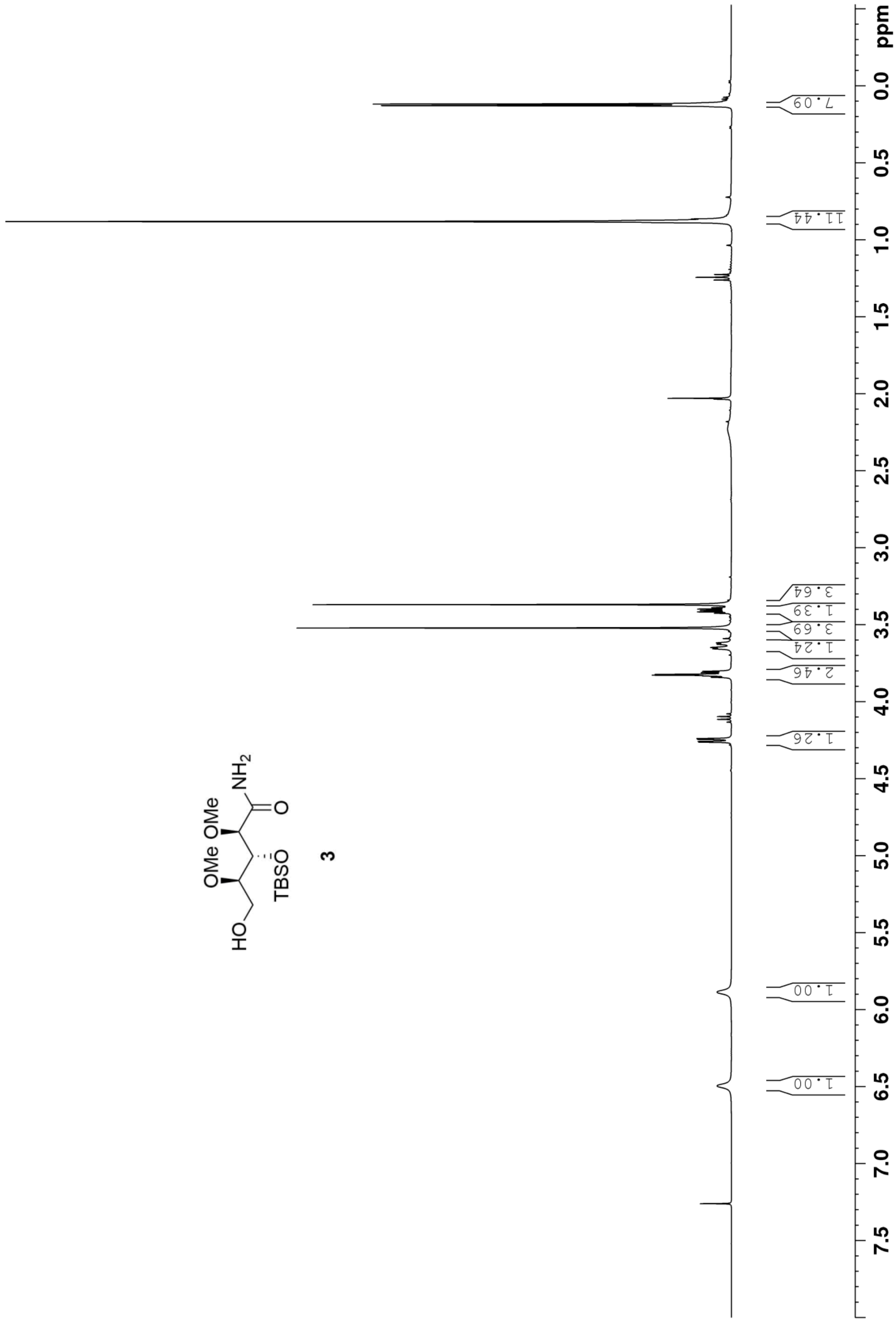
HO
OMe
OMe
NH$_2$
O
TBSO
3
ppm
0.0
0.5
1.0
1.5
2.0
2.5
3.0
3.5
4.0
4.5
5.0
5.5
6.0
6.5
7.0
7.5
7.09
11.44
3.64
1.39
3.69
1.24
2.46
1.26
1.00
1.00

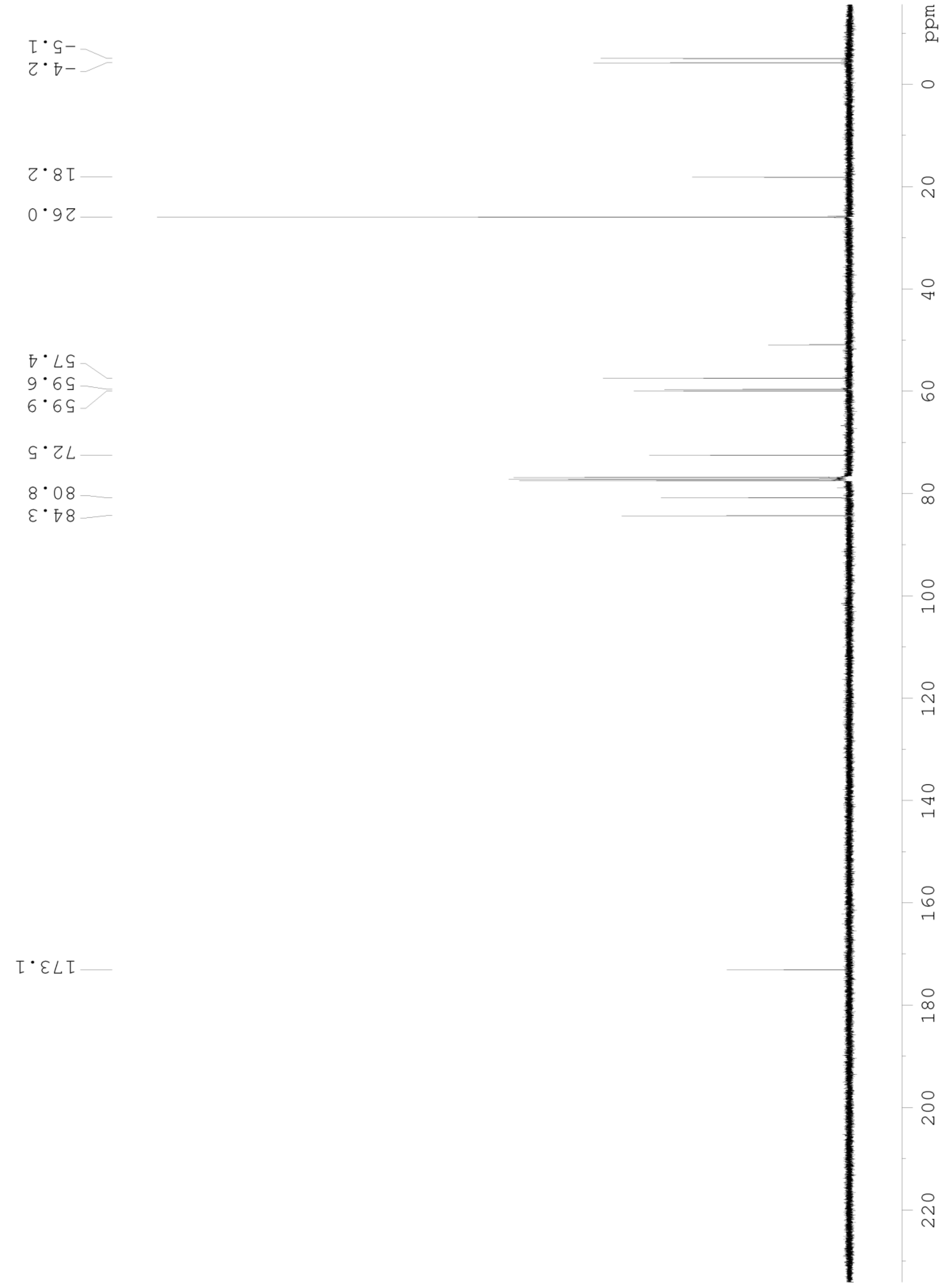

-5.1
-4.2
18.2
26.0
57.4
59.6
59.9
72.5
80.8
84.3
173.1
ppm
0
20
40
60
80
100
120
140
160
180
200
220

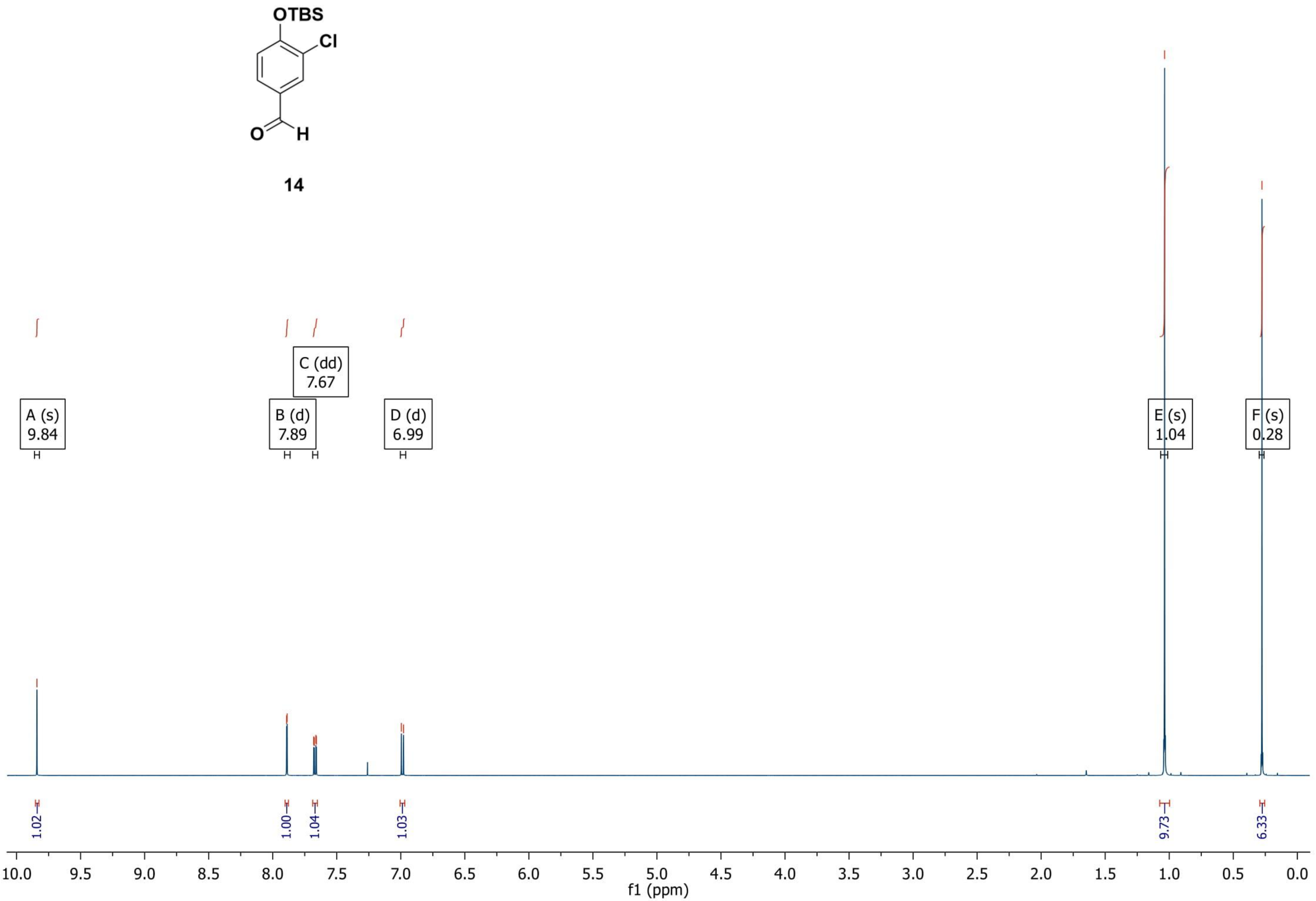

14

A (s)
9.84
B (d)
7.89
C (dd)
7.67
D (d)
6.99
E (s)
1.04
F (s)
0.28
1.02
1.00
1.04
1.03
9.73
6.33
10.0 9.5 9.0 8.5 8.0 7.5 7.0 6.5 6.0 5.5 5.0 4.5 4.0 3.5 3.0 2.5 2.0 1.5 1.0 0.5 0.0
f1 (ppm)

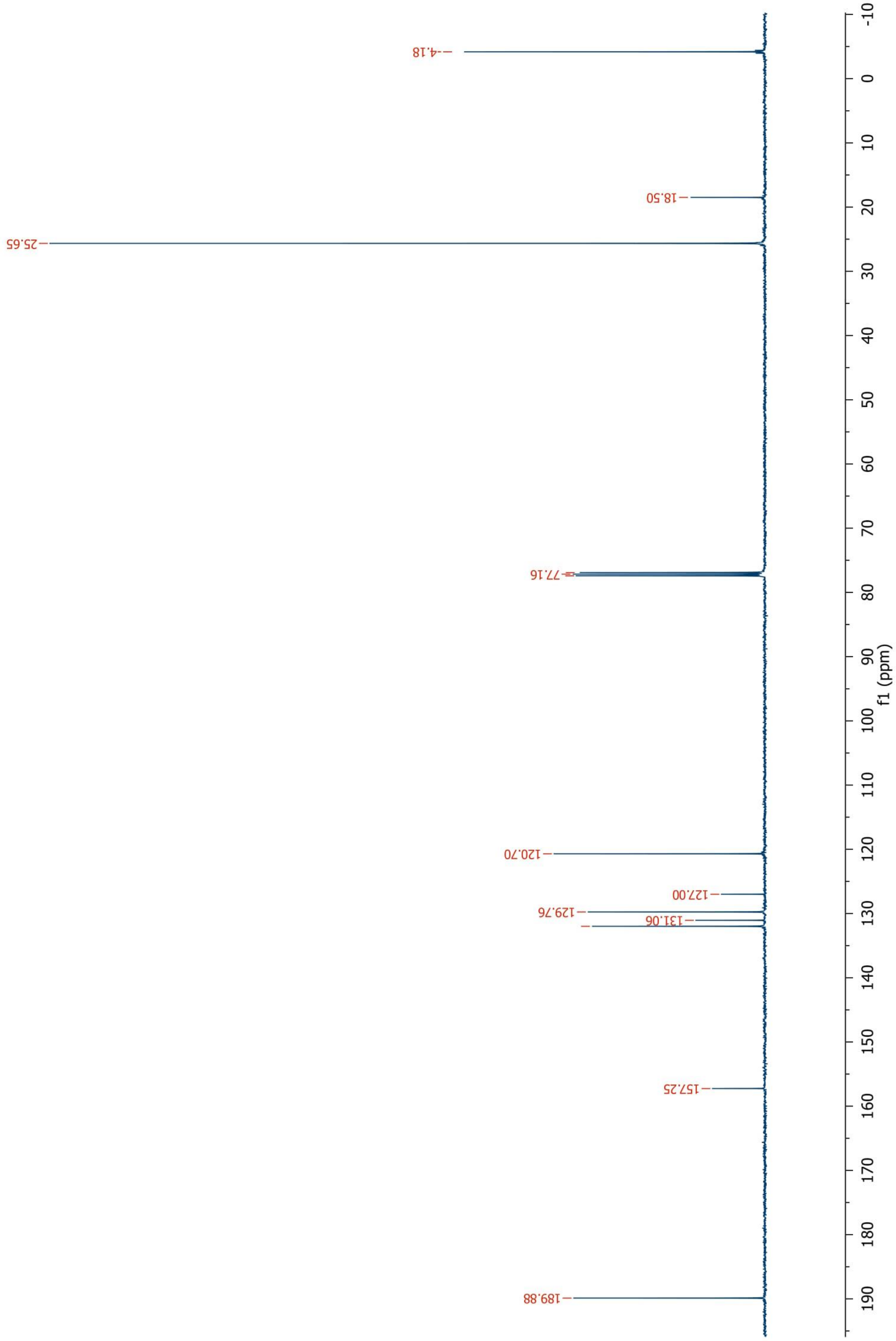

-4.18
18.50
25.65
77.16
120.70
127.00
129.76
131.06
157.25
189.88
f1 (ppm)

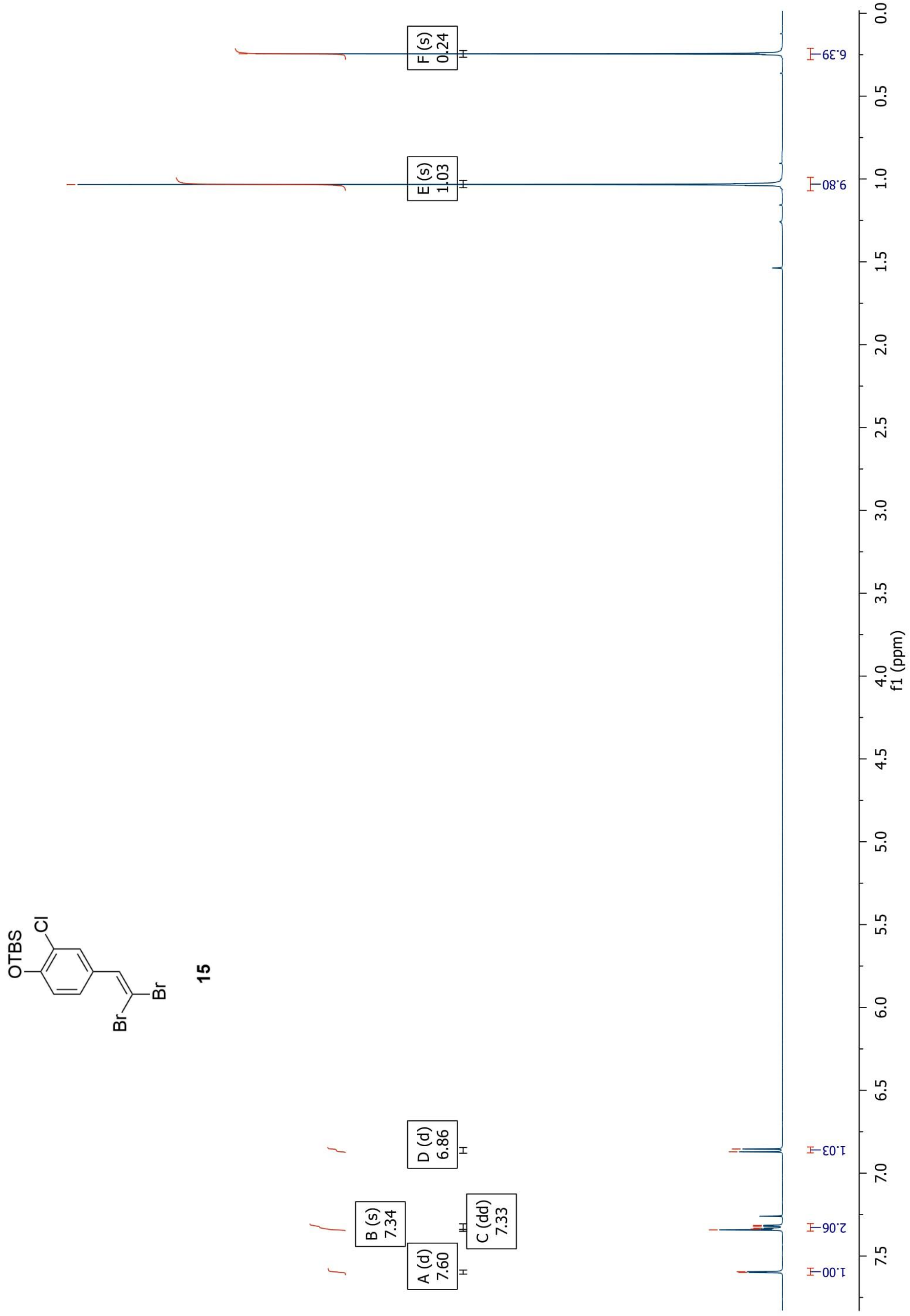
OTBS
Cl
Br
Br
15
F (s)
0.24
E (s)
1.03
D (d)
6.86
B (s)
7.34
C (dd)
7.33
A (d)
7.60
6.39
9.80
1.03
2.06
1.00
0.0
0.5
1.0
1.5
2.0
2.5
3.0
3.5
4.0
f1 (ppm)
4.5
5.0
5.5
6.0
6.5
7.0
7.5

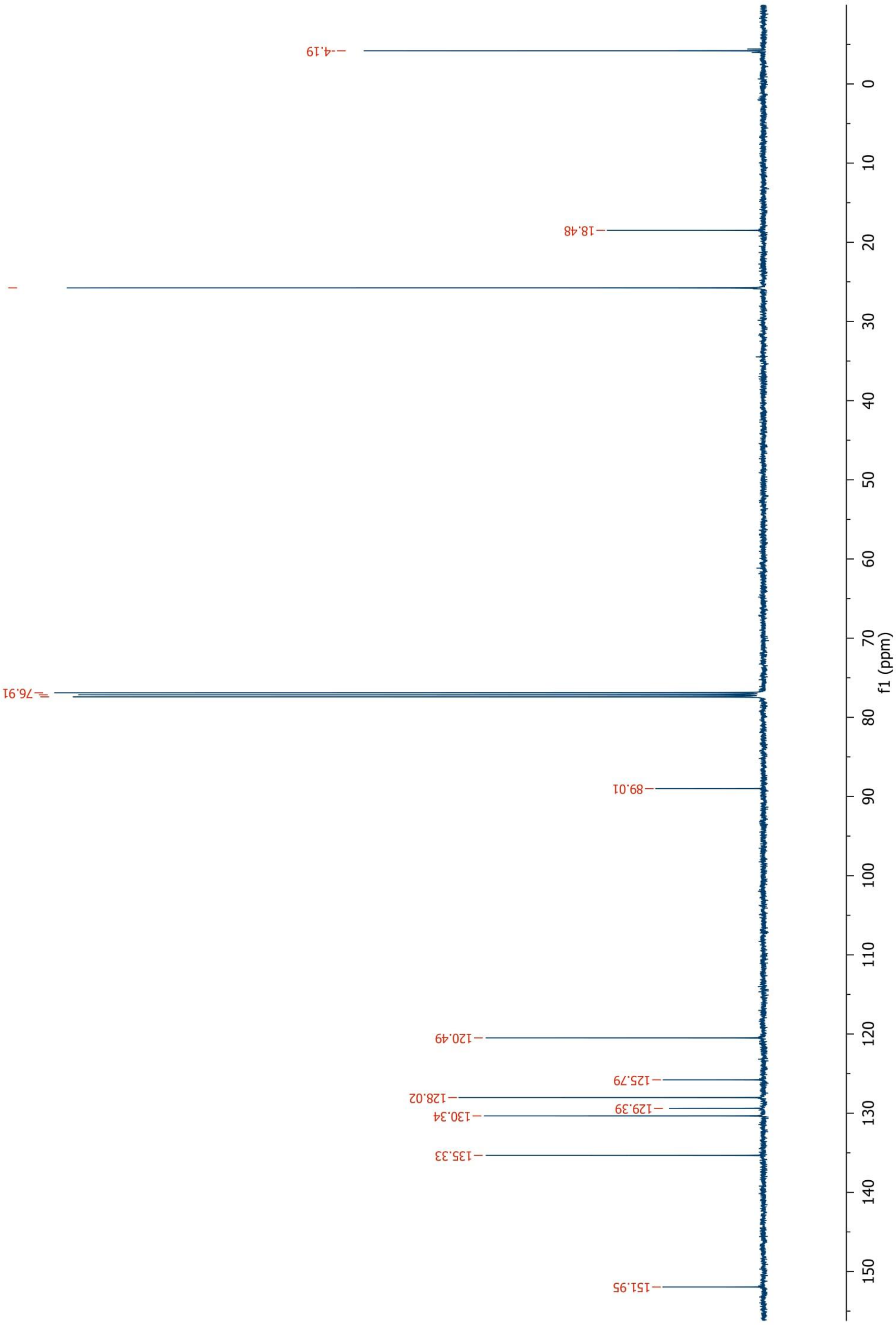

-4.19
18.48
76.91
89.01
120.49
125.79
128.02
129.39
130.34
135.33
151.95
f1 (ppm)

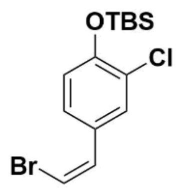

4

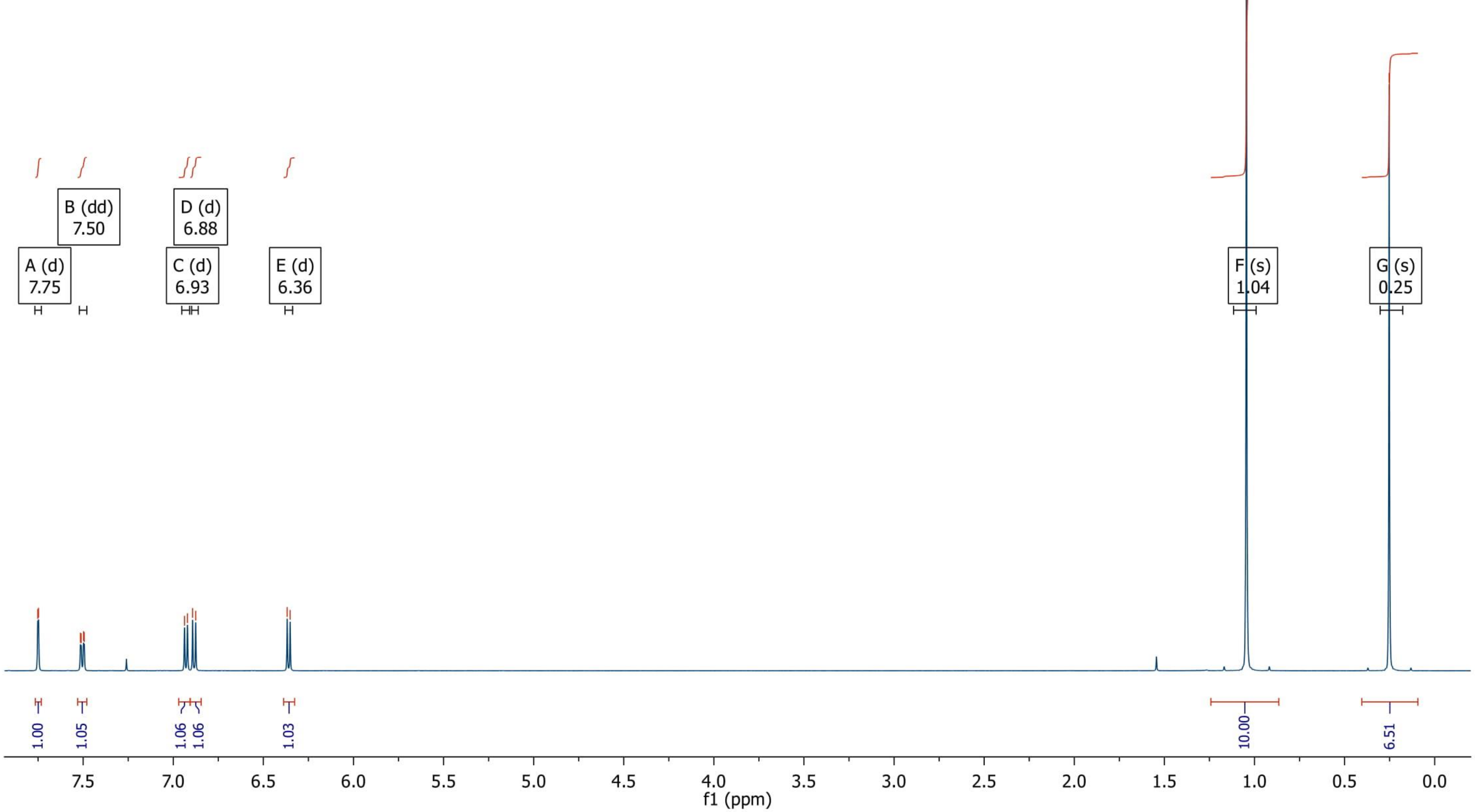

A (d)
7.75
B (dd)
7.50
C (d)
6.93
D (d)
6.88
E (d)
6.36
F (s)
1.04
G (s)
0.25
1.00
1.05
1.06
1.06
1.03
10.00
6.51
7.5
7.0
6.5
6.0
5.5
5.0
4.5
4.0
3.5
3.0
2.5
2.0
1.5
1.0
0.5
0.0
f1 (ppm)

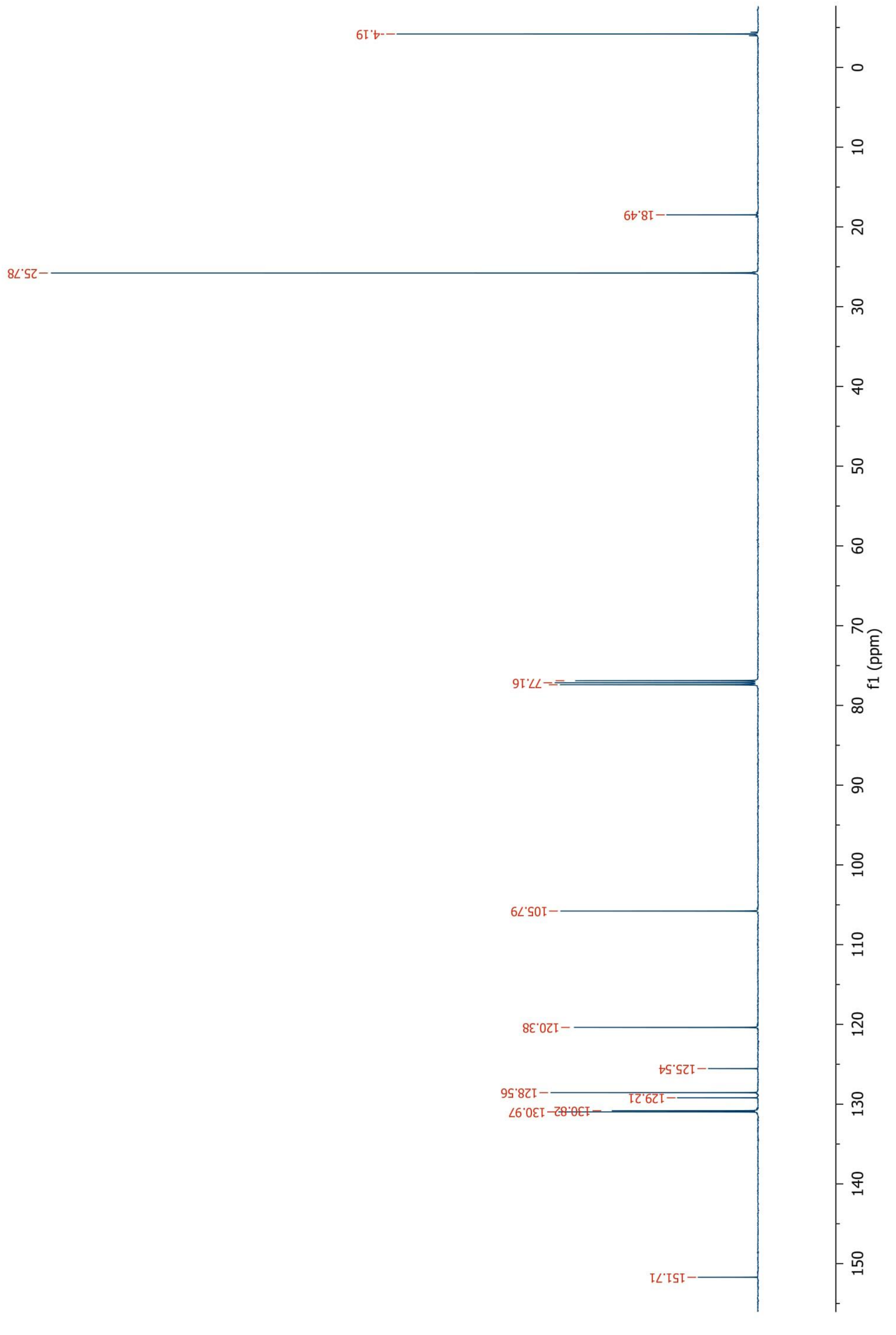
-4.19
18.49
25.78
77.16
105.79
120.38
125.54
128.56
129.21
130.82
130.97
151.71
f1 (ppm)

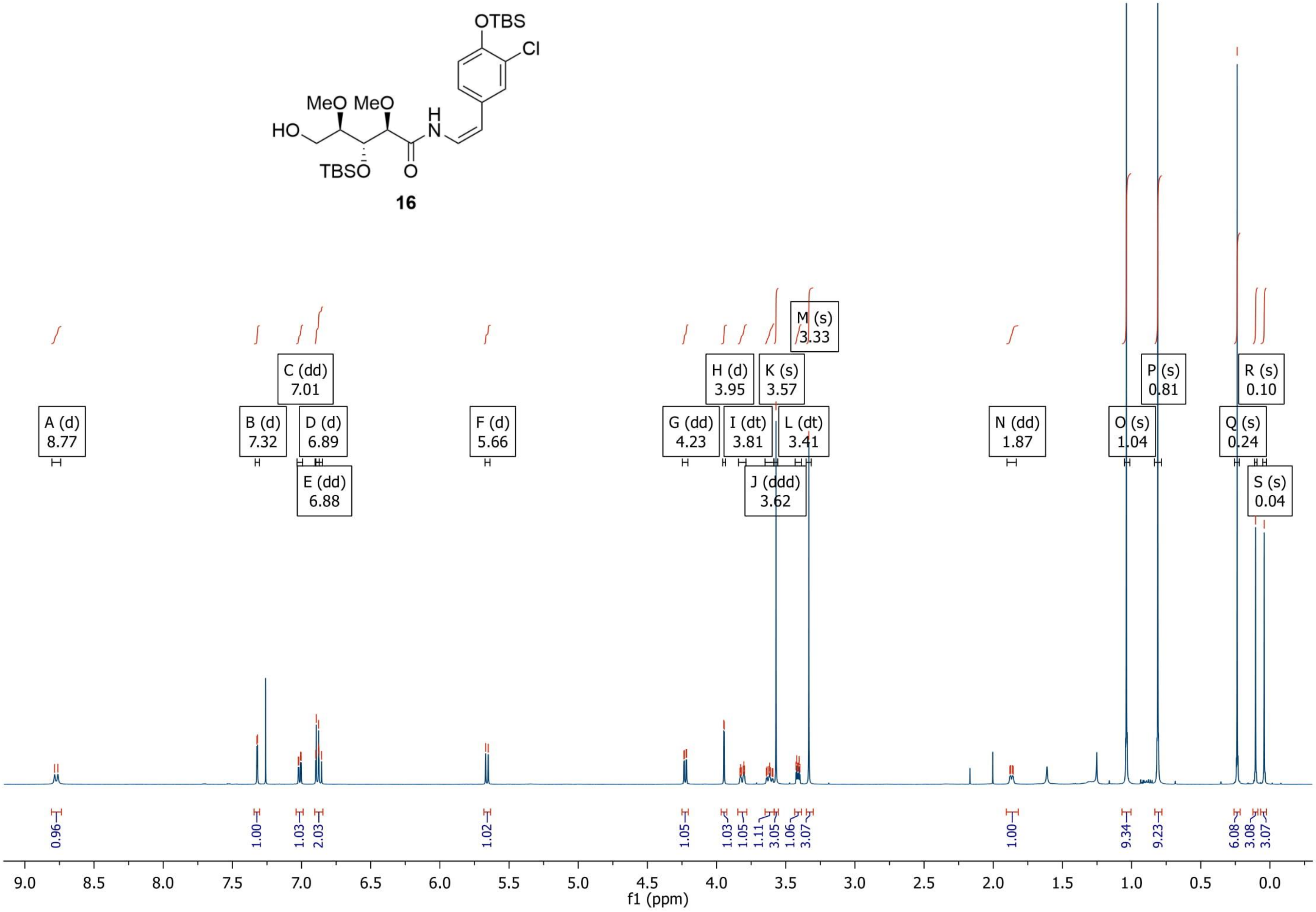
OTBS
Cl
MeO MeO
HO
H
N
TBSO
O
16
A (d) 8.77
B (d) 7.32
C (dd) 7.01
D (d) 6.89
E (dd) 6.88
F (d) 5.66
G (dd) 4.23
H (d) 3.95
I (dt) 3.81
J (ddd) 3.62
K (s) 3.57
L (dt) 3.41
M (s) 3.33
N (dd) 1.87
O (s) 1.04
P (s) 0.81
Q (s) 0.24
R (s) 0.10
S (s) 0.04
0.96
1.00
1.03
2.03
1.02
1.05
1.03
1.05
1.11
3.05
1.06
3.07
1.00
9.34
9.23
6.08
3.08
3.07
9.0 8.5 8.0 7.5 7.0 6.5 6.0 5.5 5.0 4.5 4.0 3.5 3.0 2.5 2.0 1.5 1.0 0.5 0.0
f1 (ppm)

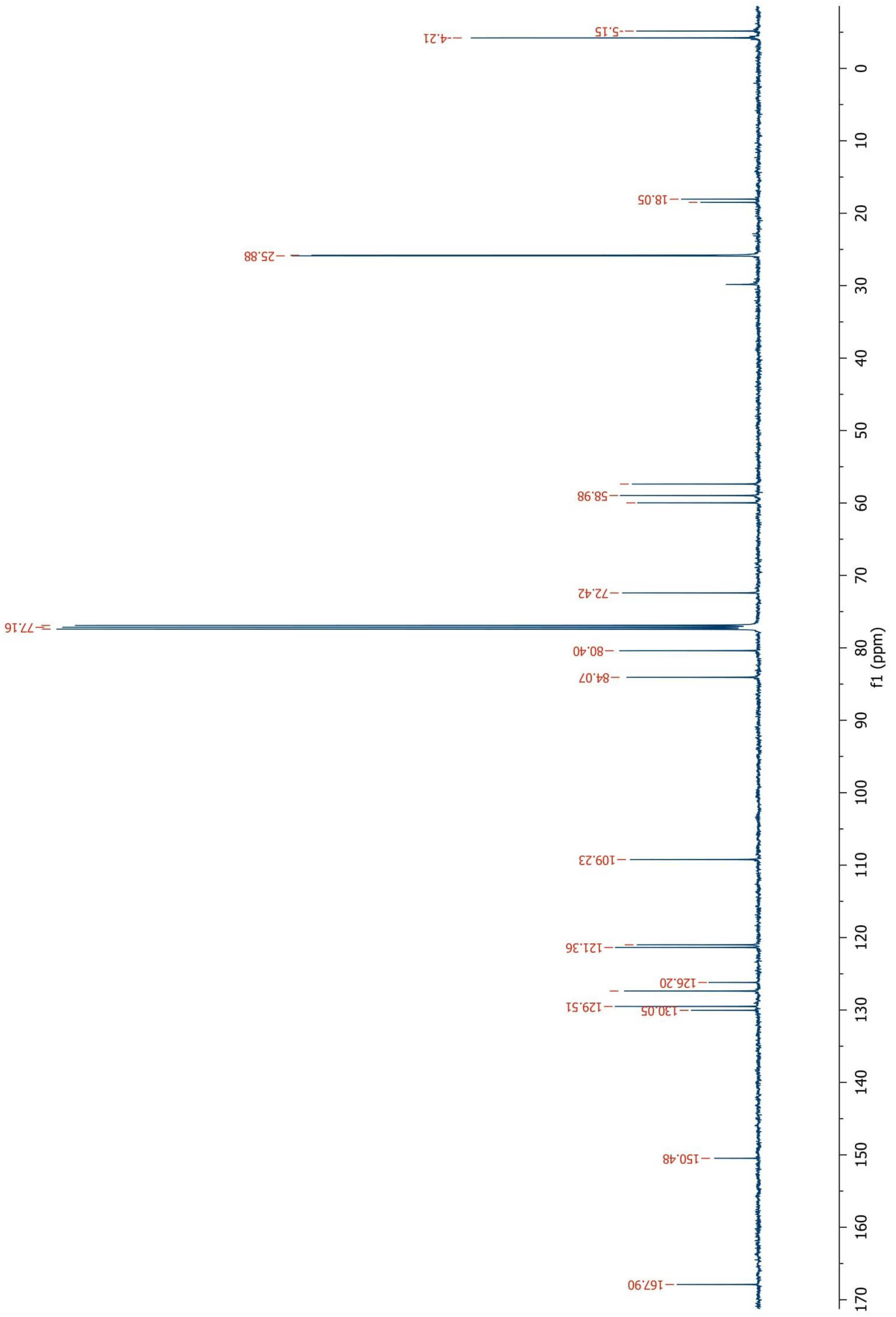

-5.15
-4.21
18.05
25.88
58.98
72.42
77.16
80.40
84.07
109.23
121.36
126.20
129.51
130.05
150.48
167.90
f1 (ppm)
